# Clinical and Preclinical Studies of Fermented Foods and Their Effects on Alzheimer’s Disease

**DOI:** 10.3390/antiox11050883

**Published:** 2022-04-29

**Authors:** Muganti Rajah Kumar, Nor Farahin Azizi, Swee Keong Yeap, Janna Ong Abdullah, Melati Khalid, Abdul Rahman Omar, Mohd. Azuraidi Osman, Adam Thean Chor Leow, Sharifah Alawieyah Syed Mortadza, Noorjahan Banu Alitheen

**Affiliations:** 1 Department of Cell and Molecular Biology, Faculty of Biotechnology and Biomolecular Sciences, Universiti Putra Malaysia (UPM), Serdang 43400, Selangor, Malaysia; drmuganti@gmail.com (M.R.K.); farahinazizi96@gmail.com (N.F.A.); janna@upm.edu.my (J.O.A.); azuraidi@upm.edu.my (M.A.O.); adamleow@upm.edu.my (A.T.C.L.); 2 China-ASEAN College of Marine Sciences, Xiamen University Malaysia, Sepang 43900, Selangor, Malaysia; skyeap@xmu.edu.my; 3 Department of Biomedical Sciences, Faculty of Medicine and Health Sciences, Universiti Putra Malaysia (UPM), Serdang 43400, Selangor, Malaysia; melati@upm.edu.my; 4 Department of Veterinary Pathology and Microbiology, Faculty of Veterinary Medicine, Universiti Putra Malaysia (UPM), Serdang 43400, Selangor, Malaysia; aro@upm.edu.my; 5 Enzyme and Microbial Technology Research Center, Universiti Putra Malaysia (UPM), Serdang 43400, Selangor, Malaysia; 6 Department of Biochemistry, Faculty of Biotechnology and Biomolecular Sciences, Universiti Putra Malaysia (UPM), Serdang 43400, Selangor, Malaysia; s_alawieyah@upm.edu.my; 7 UPM-MAKNA Cancer Research Laboratory, Institute of Bioscience, Universiti Putra Malaysia (UPM), Serdang 43400, Selangor, Malaysia

**Keywords:** Alzheimer’s disease, memory and cognition, fermented foods, probiotics, gut microbiota, oxidative stress, neuroprotection

## Abstract

The focus on managing Alzheimer’s disease (AD) is shifting towards prevention through lifestyle modification instead of treatments since the currently available treatment options are only capable of providing symptomatic relief marginally and result in various side effects. Numerous studies have reported that the intake of fermented foods resulted in the successful management of AD. Food fermentation is a biochemical process where the microorganisms metabolize the constituents of raw food materials, giving vastly different organoleptic properties and additional nutritional value, and improved biosafety effects in the final products. The consumption of fermented foods is associated with a wide array of nutraceutical benefits, including anti-oxidative, anti-inflammatory, neuroprotective, anti-apoptotic, anti-cancer, anti-fungal, anti-bacterial, immunomodulatory, and hypocholesterolemic properties. Due to their promising health benefits, fermented food products have a great prospect for commercialization in the food industry. This paper reviews the memory and cognitive enhancement and neuroprotective potential of fermented food products on AD, the recently commercialized fermented food products in the health and food industries, and their limitations. The literature reviewed here demonstrates a growing demand for fermented food products as alternative therapeutic options for the prevention and management of AD.

## 1. Introduction

Globally, dementia is one of the predominant causes of functional disability and dependency among older people (>65 years), accounting for ~50 million cases in 2020 and ~10 million new cases every year [1,2]. Data from Alzheimer’s Disease International have reported that dementia cases will be elevated to 152 million by 2050 [2], and it has accounted for 1.55 million deaths worldwide in 2019 [3]. One of the most common (60–75%) forms of dementia is AD. The two major hallmark pathologies of AD are the progressive accumulation of senile plaques, which are 37–49 amino acids long polypeptide amyloid-beta (Aβ) found in the exterior of the neurons in the brain followed by the hyperphosphorylation of neurofibrillary tangles, which are the twisted strands of tau protein found in the interior of the neurons [4]. These alterations in the brain result in progressive synaptic loss and damage and degeneration of neuronal cells leading to loss of memory, severe cognitive impairment, and inability to perform daily activities. The cause of AD is also associated with some metabolic defects such as hyperglycemia, insulin resistance, dyslipidemia, and increased biomarkers of inflammation, chronic neuroinflammation, and oxidative stress [4]. To date, there are only six specialized drugs available for treating AD, and these drugs are only capable of providing symptomatic relief marginally such as cognitive and behavioral improvement, but they do not treat the underlying causes. These include the four AChE inhibitors such as Tacrine, Donepezil, Rivastigmine, and Galantamine, Memantine the NMDA receptor antagonist, and the newly discovered Aducanumab targeting aggregated forms of Aβ [5]. These drugs often result in various side effects, including brain swelling and bleeding, cholinergic, and hepatotoxicity. Currently, there is a growing interest in the use of dietary interventions such as fermented foods as a source for highly successful disease management and drug development. The focus on the management of AD is also shifting towards prevention through lifestyle modification instead of a treatment since there is currently no treatment available to revive the degenerated brain cells. Thus, efforts to identify potential fermented food products that are safe for long-term consumption have received great attention to overcome this serious issue and are promptly needed to minimize the side effects and toxicity of the commercial drugs.

**Figure 1:**
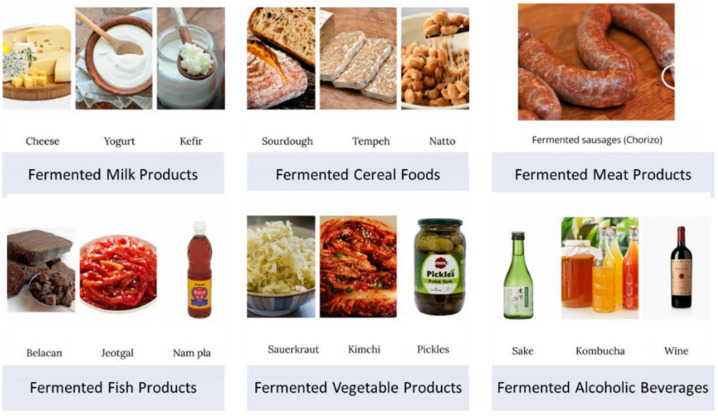
Examples of fermented foods and beverages based on divergent raw food substrates found around the world.

Fermentation is defined as a biochemical process through which the microorganisms metabolize the constituents of raw materials, giving vastly different organoleptic properties and nutritional value, as well as improved biosafety effects in the final products [6]. To date, over 5000 types of fermented foods and drinks have been discovered globally through various mixtures of foods, microorganisms, and fermentation processes (Figure 1) [7,8]. Generally, the fermented products are prepared according to the community food habits based on staple diets and the availability of plant or animal sources. In addition, each specific fermented product is unique and varies by region, culture (religions, customary beliefs, social groupings, and dietary laws), and ethnicity (race, cultural group, and people) worldwide [9,10]. Numerous studies have reported that the fermentation process enriches the nutritional contents in the food and its biological properties, including anti-oxidative, anti-inflammatory, anti-apoptotic, anti-cancer, anti-fungal, anti-bacterial immunomodulatory, and hypocholesterolemic effects, which improved health benefits [11,12,13].

This paper comprehensively reviews the neuroprotective effects of fermented foods on AD and associated symptoms, such as memory and cognition. Several studies on the potential of fermented foods and probiotic supplementation for brain and cognitive function are available [14,15,16,17]. The majority of studies, on the other hand, only looked at a few fermented foods and their link to stress and emotion-related health. They also failed to recognize the variety of their products and their importance in cognitive regulation. As a result, this review focuses on various fermented food products that have demonstrated memory, cognition, and neuroprotective effects in AD clinical and pre-clinical studies. We also discuss the probiotics included in each fermented item and how they can help with memory and cognitive problems. Many current findings are discussed in-depth, including the underlying mechanism of probiotics in the gut and their link to brain function.

## 2. Alzheimer’s Disease and Neuroinflammation

**Figure 2:**
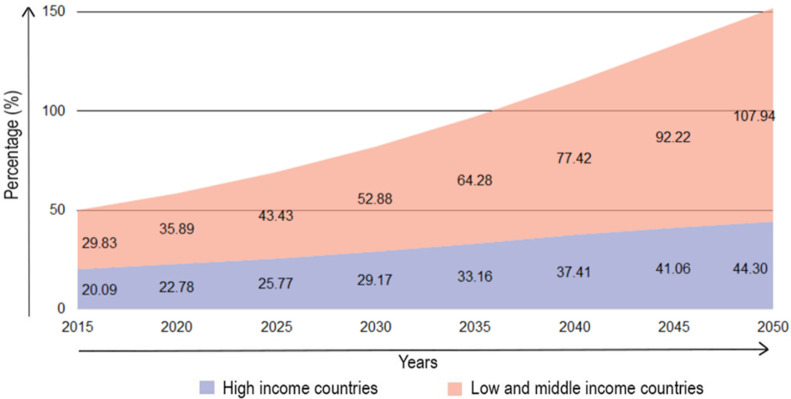
The number of people with dementia living in low and middle-income countries compared to high-income countries. “Adapted with permission from International et al. [2]. 2020, Alzheimer’s Disease International”.

The mortality rate of AD has increased tremendously compared to the mortality rate of other major diseases. The number of increased dementia cases is mostly reported in developing countries (Figure 2). Currently, about 60% of people with dementia are living in low- and middle-income countries [2]. However, this number is estimated to rise to 71% by 2050 [2]. In low- and middle-income countries, only ~5–10% of the people with dementia are diagnosed compared to the high-income countries where 40–50% of people are diagnosed [2]. As the world population moves towards older ages, the prevalence of AD and other dementias is expected to rise significantly.

AD can be classified as sporadic and familial [18,19]. Most AD cases are sporadic and it is not inherited from families. Sporadic AD is primarily due to the complex combination of genes, environment, and lifestyle. However, aging is the greatest risk factor for AD. Nonetheless, the underlying mechanism in developing sporadic AD is still unclear. On the other hand, familial AD is a rare form of AD that accounts for <1% of all AD cases [19]. It is primarily due to the mutations in specific genes that are inherited from families. To date, three types of familial AD have been discovered. The mutations can be found on presenilin 1 (PSEN1), presenilin 2 (PSEN2), and amyloid precursor protein (APP) genes, on chromosomes 14, 1, and 21, respectively [18]. It is also associated with a genetic risk factor of the APOEε4 allele [18]. Hence, familial AD can develop at any age and it often has the same symptoms as sporadic AD.

The pathophysiology of neuroinflammation is very complex and it is often driven by the activation of resident immune brain cells, particularly the glial cells such as microglia and astrocytes. In the central nervous system, glial cells are the non-excitable heterogeneous cells that are crucial for various brain functions. They often respond to brain injuries quickly and activate the repair mechanisms to restore brain physiology. Microglia is considered the first form of immune defense in the brain. They are crucial in phagocytosis, the release of cytotoxic factors, and act as antigen-presenting cells [20]. These cells are vastly distributed throughout the brain and spinal cord, retina, and optic nerve, but mainly in the substantia nigra and hippocampus. On the other hand, astrocytes are the most abundant glial cells in the central nervous system. It plays an essential role in maintaining cerebral homeostasis by regulating extracellular ion homeostasis, pH, oxidative stress, and blood flow [20]. Together with the neurons, oligodendrocytes, pericytes, and endothelial cells, these cells are accountable for the blood–brain barrier’s proper functioning. Studies from animal models and a human autopsy showed an immune response in the brain in the presence of Aβ plaques and neurofibrillary tangles and co-localization near the activated glial cells [20]. Brain injuries such as Aβ and neurofibrillary tangles deposition acquire microglia and astrocyte cells to a reactive phenotype. The reactive phenotype of glial cells causes them to lose their physiological functions (loss of three-dimensional network, morphofunctional changes, and neurovascular unit alterations) and secrete pro-inflammatory elements such as reactive oxygen species (ROS), cytotoxic elements, and cytokines leading to chronic inflammation as a protective response to eliminate the injurious stimuli [20,21]. However, prolonged and uncontrolled activation of these reactive glial cells stimulates a neuroinflammatory response. It synthesizes various cytokines and pro-inflammatory mediators, including IL-1α, IL-1β, IL-6, IL-8, IL-12, IL-18, IL-23, INF-γ, TNF-α, GM-CSF, MCP1, and MCP-113, resulting in neurodegeneration [21]. This process is known as reactive gliosis, a characteristic event of AD.

## 3. Fermented Food Products

Food fermentation is primarily attributed to preserving highly perishable food materials with a very short shelf-life, retaining nutrients, and reducing food waste. Antimicrobial compounds such as organic acid (lactic acid, acetic acid, formic acid, and propionic acid), ethanol, carbon dioxide, diacetyl, reuterin, and bacteriocins are produced by the natural microbiota of the food or by the intentional addition of starter cultures in the food fermentation process to preserve these foods [9]. Surplus milk is fermented with kefir grains, which contain a symbiotic combination of yeast and lactic acid bacteria (LAB), as part of the ancient Caucasians’ diet. As a result of these processes, they were able to store them for longer periods of time while maintaining their nutritious content. In addition, the synthesis of anti-microbial metabolites found in the kefir product, including lactic acid and other organic acids produced from LAB and ethanol produced by yeast, creates an environment that inhibits pathogens’ growth and thus protects fermented products [22]. Today, kefir (*Lactobacillus* spp.) products are recognized as highly nutritious fermented beverages with various health benefits consumed worldwide.

In addition to suppressing the spoilage of perishable foods, fermentation has been explored for other functions, such as improvement in food safety effects, enrichment in nutritional value, and development in the organoleptic quality of the food [7]. Microorganisms undergo enzymatic conversions of the fermented foods to produce several compounds. Therefore, microorganisms have been responsible for determining the course and outcome of fermentation processes, which contribute to developing the characteristic properties of the final fermented food [23]. For instance, during lactic acid fermentation, LAB produces organic acids, primarily lactic acid, which is the characteristic fermentative product and reduces the pH of the substrate to a level where the growth of pathogenic, putrefactive, and toxigenic bacteria is inhibited [24]. Moreover, microorganisms also release hundreds of metabolites, including glycerol, acetic acid, hydrogen sulfide, and phenolic acids, enhancing the aroma and flavor of a product, and making it palatable [25,26]. For instance, Marullo and Dubourdieu [27] demonstrated that yeast strain selection determined the concentration of aromatic molecules and the organoleptic perception of wine. Additionally, Walther and Schmid [28] also reviewed the effect of fermentation on vitamin content in food, such as riboflavin, vitamin B12, folate, and vitamin K. The study shows that different nutritional content can significantly and naturally be increased in fermented food, which consequently provides a solution to overcome food and nutrition insecurity.

The most common microorganisms involved in food fermentation are bacteria, yeasts, and molds. Microorganisms involved in the fermentation of various products, including traditional and novel fermented foods, have been reviewed extensively [6,7,9,22,24,29,30,31,32,33]. Briefly, bacteria (primarily of the LAB group) are the dominant microorganisms with lactic acid as the primary end-product during fermentation. *Lactobacillus* is the most prevalent genus in fermentation during the production of acidic fermented products. Examples include fermented cereals, kimchi (fermented and spiced Napa cabbage from Korea), sauerkraut (fermented cabbage from eastern and central Europe), yogurt, sausages, and cheese. Other important bacteria, including *Bacillus*, can be commonly found in the fermentation of legumes, including rabadi (fermented maize from Mexico), kishk (fermented wheat from the Middle East), fermented rice, and fermented soybean [24,34]. Yeasts are predominant in the microbial composition of much alcoholic food fermentation, mainly for baking and brewing (wine, beer, leavening of bread), which lead to the release of ethanol and carbon dioxide [35,36]. Molds belong to the third group of microorganisms have some main functional properties in fermented foods, including the production of enzymes (α-amylase, acid/alkaline proteases, amyloglucosidase, β-galactosidase, cellulase maltase, hemicellulase, invertase, lipases, pectinase), and the deterioration of anti-nutritive factors, or enhancing the bioavailability of minerals [37]. For example, Joshi et al. [38] showed species of *Aspergillus* are involved in the production of pectinase from apple pomace, which opens several applications in food security due to its low-cost raw materials.

Fermented foods have been linked to significant health benefits. Plentiful populations of living microbial cultures and metabolites may add to the functional qualities of fermented food, according to various studies. These protective effects may be related to increased bioavailability of particular nutrients (protein, vitamins) or decreased anti-nutritional chemicals (phytates, tannins, polyphenols) due to fermentation-induced microbial growth and enzymatic conversions of food components [8]. Marco et al. [23] proposed that fermented foods could benefit health by modulating the immune system and gut microbiota composition and activity by altering intestinal and systemic function. Studies on the health benefits of fermented foods have been reviewed recently [7,9,32,39,40]. Lavefve et al. [31] examined the health advantages of fermented foods and beverages, including coffee. Fermented grains and legumes also provide health benefits [24]. Table 1 lists some fermented foods’ health-promoting properties.

Fermented products’ antihypertensive effect depends on angiotensin-converting enzyme inhibitory (ACE-I) peptides. They prevent angiotensin II from becoming angiotensin I, causing hypertension. As well as bradykinin and kallidin, ACE inhibits the risk of cardiovascular diseases (CVD). For example, *Lactobacillus rhamnosus* fermented camel milk showed impressive ACE-I peptides, indicating its potential application in lowering blood pressure in mildly hypertensive individuals [49]. Another peptide compound with a hypotensive effect is Gamma-aminobutyric acid (GABA). For example, the spontaneously hypertensive rat and Wistar Kyoto rat both had significantly lower systolic and diastolic blood pressure after administration of fermented beans by *Bacillus subtilis* strain B060 [55].

The antimicrobial properties of fermented foods, including organic acids and bacteriocins, CO_2_, hydrogen peroxide, ethanol, and diacetyl, suppressed pathogen growth. Similarly, five bacteriocin-producing lactic acid bacteria (*Lactobacillus plantarum* ST69BZ, *Enterococcus faecium* ST62BZ, and *Leuconostoc lactis* ST63BZ, ST611BZ, and ST612BZ) in boza (fermented cereal-based from Bulgaria) showed bactericidal effects against *Enterococcus* spp., *Escherichia coli*, and *Klebsiella pneumoniae* [58]. Additionally, phytase activity was tested during bread making by yeast for reduction of anti-nutritive activity, and retained minerals such as calcium and iron [70].

Free radicals are also recognized to have important roles in cell signaling, apoptosis, ion transport, and gene expression. However, severe oxidative stress can cause degenerative diseases like cancer, CVD, Alzheimer’s, and Parkinson’s. The body can regenerate non-enzymatic (reduced glutathione (GSH)) and enzymatic antioxidants (SOD, GSHPx, and CAT). However, natural antioxidants may not be sufficient to maintain good cellular function, necessitating exogenous antioxidants [71]. Fermented foods have been shown to produce free amino acids, free polyphenols, genistein and daidzein (isoflavones), malvidin, delphinidin (flavonoids), and aglycones [7,72]. For example, fermented soy whey had 4.5 times more daidzein and genistein than unfermented soy whey [66]. Fermented foods have developed beyond preservation and are now marketed as “super-foods” for their functional health-promoting effects.

## 4. Clinical Studies

To date, there are only a few clinical studies have been reported regarding the effects of fermented foods on AD, as shown in Table 2. Hence, studies on the impact of fermented foods on typical AD symptoms such as memory and cognitive impairment were also discussed in this review.

Probiotics are found in various fermented foods. Probiotics from fermented foods and foods fermented with probiotics are well-known to have many health benefits, including neuroprotective potential. Kefir is a self-carbonated and refreshing fermented drink that has been consumed for over a hundred years for its various health benefits. Fermentation of kefir requires kefir grains which are comprised of various microorganisms, particularly LAB, acetic acid bacteria, and yeasts coexisting in a symbiotic relation [82]. A recent study by Ton et al. [73] revealed that the continuous supplementation of milk kefir improved cognitive and metabolic disorders in AD patients. It was an uncontrolled clinical study carried out to investigate the effects of milk kefir supplementation (2  mL/kg/daily) for 90 days in AD patients with cognitive deficits. The study assessed the cognitive function, the levels of systemic oxidative stress, cytokine expression, and blood cell damage biomarkers before and after milk kefir supplementation. The study demonstrated that most AD patients supplemented with milk kefir exhibited a marked improvement in memory, executive/language functions, and visual-spatial/abstraction abilities. The cytometry analysis showed a ~30% reduction in the inflammation and oxidative stress markers (O_2_^−^, ONOO^−^, and H_2_O_2_) accompanied by a 100% increase in NO bioavailability. Additionally, improvement in serum protein oxidation, apoptosis, DNA damage/repair, and mitochondrial dysfunction [83,84,85] were reported. The study indicated that the milk kefir supplementation improves cognitive deficits and the factors associated with AD such as oxidative stress, systemic inflammation, and blood cell damage.

A study was designed by Chung et al. [74] to assess the effects of *Lactobacillus helveticus* IDCC3801 fermented in processed skim milk powder (LHFM) on cognitive function in healthy older adults. The study conducted a 12-week randomized, double-blinded, and controlled experiment. Several tests were performed before and after the experiment: Cognitive tests such as neuropsychological and cognitive fatigue, and measurements of the geriatric depression scale-short form (GDS-SF), perceived stress scale (PSS), whole blood viscosity (WBV), and brain-derived neurotrophic factor (BDNF). The study showed that the supplementation of LHFM for 12 weeks in healthy older adults improved cognitive function. The study found no significant effects on GDS-SF, PSS, WBV, and BDNF. Similarly, Ohsawa et al. [75] studied the effects of supplementation of *Lactobacillus helveticus* fermented milk containing lactononadecapeptide (NIPPLTQTPVVVPPFLQPE) on the cognitive function of healthy Japanese middle-aged adults. It was a double-blinded, randomized, controlled study where participants were randomly assigned to receive the *Lactobacillus helveticus* fermented milk supplementation (190 g/day) for 8 weeks. The cognitive function of the participants was examined using the Japanese version of the repeatable battery for the assessment of the neuropsychological status (RBANS) test. The study indicated a significant improvement in attention and delayed memory in participants supplemented with *Lactobacillus helveticus* fermented milk. Hwang et al. [76] also reported the positive effects of probiotic supplementation, *Lactobacillus plantarum* C29-fermented soybean (DW2009), on individuals with mild cognitive impairment. The participants were randomly assigned into two groups: DW2009 group (*n* = 50) receiving 800 mg/day of the probiotic supplementation or the Placebo group (*n* = 50). The study evaluated the neurocognitive function, measurement of serum BDNF levels, and fecal microbiota analysis. The study reported that the participants supplemented with DW2009 had shown greater improvement in their cognitive functions, particularly in the attention domain. The improved cognitive function was found to be associated with increased BDNF levels. The study also found a significant increase in the number of lactobacilli in the gut bacterial composition of the participants supplemented with DW2009, suggesting the importance of the gut–brain axis in mitigating cognitive deficits in individuals with mild cognitive impairment.

A study by Akbari et al. [77] has found that a daily dose of fermented dairy products (probiotic supplementation) improved cognitive function in older AD patients. The study was a randomized, double-blind, controlled trial with 60 older AD patients at 60–95 years of age. The patients were randomly divided into two groups, where one group received probiotic supplementation while another received milk (control group) for 12 weeks. Each of the probiotic supplemented groups received 200 mL/day of probiotic milk containing 2 × 10^9^ CFU/g of *Lactobacillus acidophilus*, *Lactobacillus casei*, *Lactobacillus fermentum*, and *Bifidobacterium bifidum*. The analysis of pre- and post-treatment mini-mental state examinations (MMSE) and fasting blood samples were collected. The results showed significant improvement in the MMSE score for the patients treated with probiotic supplementation. Additionally, in the probiotic treated group, the changes in the plasma malondialdehyde, beta-cell function, serum high-sensitivity C-reactive protein, serum triglycerides, homeostasis model of assessment-estimated insulin resistance, and quantitative insulin sensitivity check index were significantly varied when compared to the control group. The study suggested that the probiotic supplementation for 12 weeks improved cognitive function and some metabolic statuses in the older AD patient.

Studies have reported that fermented and unfermented soybean products such as soy milk, tofu (soybean curd), tempeh, soy sauce, and miso are associated with a reduced risk of cognitive impairment. However, the effects of the fermented soybean products on memory and cognitive function were reported to be greater than the unfermented soybean products, proving the potential effects of fermentation [78,86]. Hogervorst et al. [86] suggested that the high consumption of tofu (soybean curd) among the older people (52–98 years old) living in Borubudur, Sumedang, and Jakarta have been shown to worsen their memory. In contrast, high tempeh consumption in participants over 68 years of age have been shown to have a better memory. The study suggested that the presence of phytoestrogen in tofu may be associated with memory loss among the older people of Indonesia. Tempeh also contains high levels of phytoestrogens. However, the fermentation of tempeh produces high folate contents, which exert protective effects. Thus, the consumption of tempeh that contains folate did not affect the memory of older people. A follow-up study by Hogervorst et al. [78] with the older people of Borubudur, who have participated in a previous study by Hogervorst et al. [86] in 2008, demonstrated that the dietary and lifestyle changes have positively affected the health status among young and middle-aged people, but not older people. The study suggested that the estrogenic compounds did not affect the memory and cognitive function of young and middle-aged people.

In addition, a study by Handajani et al. [79] reported on the influence of the amount of tempeh consumption or the required amount of microorganisms found in tempeh serving per day on cognitive functions. The study was carried out with 90 participants aged 60 years or over with mild cognitive impairment. The participants were divided into three groups: Group A consuming 100 g of Tempeh A/day, Group B consuming 100 g of Tempeh B/day, and Group C as control. The tempeh was consumed every day for 6 months. The assessments for cognitive function were carried out before and after the intervention. At the end of the intervention, blood uric acid level was checked to examine the effect of tempeh. The study showed increased global cognitive scores in both Tempeh A and B consumed participants. However, the study reported an increase in language domain scores which was only found in Tempeh A consumed participants. Overall, Tempeh A or Tempeh B consumption, which had a lower number of microorganisms and a high number of microorganisms (Enterobacteriaceae and lactic acid bacteria), respectively, improved the global cognitive functions in older people with mild cognitive impairment. The study suggests that the greater effects of Tempeh A on global cognitive and language function might be due to the type of bacterial species or that the present microorganism may play a role in stimulating cortical grey and white matter neuroplasticity.

Moreover, Ozawa et al. [80] reported the association between dietary patterns in a general older Japanese population and the risk of dementia. The study investigated 1006 older Japanese people aged 60–79 years without dementia. Follow-up studies were carried out for a median of 15 years. The dietary patterns of the older Japanese population were determined using the reduced rank regression procedure. A Cox proportional hazards model computed the estimated risk of the development of dementia conferred by a particular dietary pattern. The study showed that higher adherence to a dietary pattern such as increased consumption of soybean and soybean products, algae, green and other vegetables, and milk and dairy products and reduced consumption of rice was associated with reduced risk of all-cause dementia, AD, and VaD in the older Japanese population.

In addition to fermented probiotics, dairy, and soybean products, fermented fruit such as papaya (Carica papaya Linn) also has beneficial impacts on AD. Barbagallo et al. [81] investigated the effects of fermented papaya powder extracts (Carica papaya Linn fermentation with yeast) on the level of oxidative stress in patients with AD. The study recruited 40 patients at the mean age of 78 years: 28 were diagnosed with early mild AD, and 12 were control patients. Out of 28 AD patients, 20 received a 4.5 g/day of the fermented papaya extracts for 6 months, while the other 8 AD patients did not receive any treatment. The level of oxidative stress was assessed by measuring the amount of 8-OHdG in urine. The study reported a significant increase in 8-OHdG in AD patients compared to the control. The study found that the administration of the fermented papaya extracts in AD patients significantly decreased the level of 8-OHdG, indicating its potential effects in alleviating the excessive production of ROS [87,88] in patients with AD.

## 5. Preclinical In Vivo Studies

Table 3 summarises the findings from preclinical in vivo studies on fermented food consumption and its effects on cognitive function and memory.

Aβ, amyloid-beta; BDNF, brain-derived neurotrophic factor; GDNF, glial cell-derived neurotrophic factor; SABT, spontaneous alteration behavior test; NORT, novel object recognition test; PAT, passive avoidance test; MWMT, Morris water maze test; OFT, open field test; IGF-1, Insulin-like growth factor 1; AChE, acetylcholinesterase; ACh, acetylcholine; MDA, malondialdehyde; SOD, superoxide dismutase; NGF, nerve growth factor; ROS, reactive oxygen species; TBARS, thiobarbituric acid reactive substances; ApoE, apolipoprotein E; sAPPα, soluble amyloid precursor protein; HSPA1A, heat shock protein family A (Hsp70) member 1A; Nrf2, nuclear factor erythroid 2-related factor 2; p-JNK, phosphorylation c-Jun N-terminal kinase; NF-ĸβ, nuclear factor kappa B; TNF-α, tumor necrosis factor-alpha; MAPK, mitogen activated protein kinase; ACAT, acyl-coenzyme A: cholesterol acyl transferase; CBS, cystathionine beta synthase; GSH, glutathione; GPx, glutathione peroxidase; CREB, cyclic AMP response element-binding protein; iNOS, inducible nitric oxide synthase; COX-2, cyclooxygenase-2 or prostaglandin-endoperoxide synthase 2; HO-1, heme oxygenase-1; Iba-1, ionized calcium binding adaptor molecule 1; ALT, alanine aminotransferase; AST, aspartate aminotransferase; NO, nitric oxide; BACE-1, beta-secretase 1; APP, amyloid precursor protein.

### 5.1. Fermented Dairy Products

Dairy products have been consumed since 9000 B.C. for their nutritional value and health benefits. Traditional fermented dairy products have evolved into a healthy diet choice in practically every country. Recent research suggests that fermented dairy products may help prevent and treat AD [134,135]. Moreover, previous research has revealed that fermented dairy products are neuroprotective [134,136].

In a 5xFAD AD mice model, *Penicillium candidum* fermented camembert cheese reduced Aβ buildup, decreased hippocampus inflammatory cytokine production (MIP-1α and TNF-α), and increased hippocampal neurotrophic factors BDNF and GDNF [89]. The preventative agent found in *Penicillium candidum* fermented camembert cheese was oleamide, a unique dual-active component. Microglial phagocytosis of Aβ was improved by oleamide, which may be a dementia prevention strategy. Liu et al. [90] found that oral administration of *Lactobacillus plantarum* strain TWK10 fermented soymilk improved learning and memory and lowered blood pressure in deoxycorticosterone acetate-induced VaD rats. The study concluded that regulating oxidative stress and blood pressure improved learning capacity in rats and reduced dementia incidence. Similarly, Ohsawa et al. [91] found that administering scopolamine-impaired ddY mice *Lactobacillus helveticus* fermented milk (Calpis sour milk whey) improved object recognition memory and scopolamine-induced cognitive impairments. 

Other neuroprotective properties of fermented dairy products were explored in aged mice by Ano et al. [92]. They digested the fermented dairy products and screened for peptides that could improve memory in scopolamine-induced amnesia mice. The whey protein Trp-Tyr (WY) peptides improved memory performance in aged mice. The WY-containing peptides raised dopamine and monoamine levels in brain tissue by blocking monoamine oxidase-B activity, preventing age-related cognitive decline. Ano et al. [93] investigated the effects of WY-containing peptides on brain microglial activation and cognitive impairment in aged and AD rats. WY peptides improved cognitive impairment in AD rats by increasing microglial Aβ phagocytosis and decreasing microglial inflammatory responses. Oral WY peptides were easily absorbed into the circulation, supplied to the brain, and reduced cognitive deterioration in LPS-induced mice. The findings imply that WY peptides’ ability to regulate microglial activity may be used to prevent dementia and cognitive decline.

Liu et al. [94] studied Tibetan fermented milk’s effect on memory impairment in APP/PS1 transgenic mice. Tibetan fermented milk reduced cognitive impairment in APP/PS1 mice, including object recognition/memory and spatial learning/memory. 16S rRNA sequencing of APP/PS1 mice feces indicated increased gut microbial diversity and relative abundance of *Bacteroides* and *Faecalibacterium* spp. Tibetan fermented milk reduced Aβ deposition in the hippocampus and cortex of APP/PS1 mice. The study found that long-term Tibetan fermented milk supplementation improved the microbial flora composition and cognitive functions in APP/PS1 mice. This discovery may help prevent and treat AD-induced cognitive decline. In addition, a study evaluated the effects of long-term consumption of β-lactolin (found in fermented dairy products) and whey digestion on 5FAD transgenic and PS19 tauopathy mice [95]. It was found that supplementing 5FAD transgenic mice with whey digestion rich in β-lactolin reduced inflammatory cytokine levels, Aβ levels, activated microglial infiltration, and improved long-term object memory impairment while increasing dopamine, synaptophysin, and BDNF levels in the cortex. In PS19 tauopathy mice, β-lactolin and whey digestion rich in β-lactolin reduced phosphorylated tau to total tau ratio in the cortex and improved behavioral abnormalities. Long-term ingestion of β-lactolin suppresses inflammation and mitigates cognitive impairment and AD pathology.

### 5.2. Kefir

Dysbiosis is defined as a loss of microbial diversity or gut microbiota malfunction. Dysbiosis has been linked to oxidative stress, inflammation, neurotransmitter depletion, apoptosis, and insulin resistance, all of which contribute to the beginning of AD [108,137,138]. Kefir, a probiotic-rich fermented drink, has shown promise in treating cognitive decline and AD. Batista et al. [107] investigated the amyloidogenic pathway in Drosophila melanogaster, an AD-like fly. The study showed increased Drosophila melanogaster’s climbing ability, survival rate, neurodegeneration index, and reduced vacuolar lesions with the n-butanol fraction of kefir treatment. The study reveals that kefir and its fraction may be a suitable therapy source for AD. Similarly, El Sayed et al. [108] investigated the effects of a kefir product, Probiotics Fermentation Technology (PFT), on AD symptoms in streptozotocin-(STZ-)-induced AD rats. PFT supplementation reduced neuronal degeneration, increased ACh levels, and improved memory and cognition in STZ-induced rats. PFT supplementation also reduced pro-inflammatory cytokine expression, oxidative damage, apoptosis, Aβ accumulation, and tau hyperphosphorylation. The study found that PFT can reduce AD symptoms by regulating the gut microbiota and preventing AD-related pathological processes.

Ali et al. [109] studied milk kefir’s benefits in LPS-induced AD albino rats. In LPS-induced rat brains, milk kefir enhanced BDNF, Bcl-2, and seladin-1 expressions, lowered oxidative stress and decreased Aβ and tau pathology. Moreover, rats spent less time in the T-maze after consuming kefir, showing strong memory recovery. Anwar et al. [110] also found that the milk kefir treatment increased CBS expression and GSH levels while decreasing Aβ, tau, MAPK, ACAT, and MDA levels in brain tissue of LPS-induced rats. The study found that the use of milk kefir as a neuroregenerative treatment reduced the pathology markers of AD while preserving inflammatory brain factors.

### 5.3. Fermented Legumes and Cereal-Based Products

Worldwide, legumes and cereals are important protein and carbohydrate sources. Rice and soybeans are the key ingredients in fermented legumes and cereal-based foods. Fermented legumes and cereals contain bioactive compounds that are absent in unfermented foods. Fermented soybean products are most popular in Asian countries. These include tofu, tempeh, soy sauce, and soybean pastes (doenjang and miso).

Cheonggukjang, a traditional Korean fermented food made by short-term fermentation of soybeans using a mixed culture of *Bacillus subtilis* and *Lactobacillus sakei* revealed neuroprotective benefits. The Cheonggukjang pre-treatment for 4 weeks significantly improved the trimethyltin-induced short- and long-term memory loss [96]. Co-treatment of Cheonggukjang and trimethyltin demonstrated fewer dead cells in the dentate gyrus granule cell layer, significant decrease in AChE [139,140], and Bax/Bcl-2 levels, and an increase in NGF [141,142] concentration and SOD activity [96]. In Aβ-induced diabetic rats fed a high-fat diet, Yang et al. [97] investigated the protective effects of traditionally fermented Cheonggukjang (TFC) and standardized Cheonggukjang (SFC) fermented with *Bacillus licheniformis* SCD 111067P. SFC increased isoflavone aglycones, E soyasaponin Be, DDMP soyasaponin βg, and lysophosphatidylcholine contents reduced Aβ buildup, and improved insulin signaling, cognition, and glucose regulation in AD diabetic rats. 

Red mold rice (RMR) is rice fermented with Monascus, red yeast. RMR is a staple in China. It has been used for generations to enhance food flavor and color, as well as for vascular and digestive health [143,144]. RMR contains monacolins, phenol, dimerumic acid, sterol, tannin, azaphilones, monounsaturated fats, and furanoisophthalides [145]. These bioactive compounds provide antioxidants and anti-inflammatory activities to effectively protect the brain [145]. Lee et al. [98] investigated the effects of RMR on AD risk factor expression, memory, and learning ability in intracerebroventricular Aβ40-induced hyperlipidemic rats. Passive avoidance and water maze tasks demonstrated that RMR improved memory deficit. RMR treatment reduced brain cholesterol levels via hypolipidemic agents, reducing oxidative stress and lipid peroxidation. By inhibiting cholesterol-mediated ApoE expression, β-secretase activity, and APP proteolysis, the RMR effectively inhibits Aβ production and deposition, resulting in neuroprotective sAPPα secretion in the hippocampus.

Kurozu is a steamed rice vinegar from Japan. Fermenting and aging Kurozu liquid for over a year yields Kurozu Moromi [99]. Kurozu and Kurozu Moromi are antioxidants and neuroprotectants. A study by Kanouchi et al. [99] examined the effects of concentrated Kurozu and Kurozu Moromi on cognitive impairment in aged P8 mice. Kurozu reduced Aβ accumulation and cognitive impairment and enhanced HSPA1A expression in the mice brain. Kurozu Moromi exhibited a slight but non-significant improvement in cognitive impairment. The data reveals that concentrated Kurozu may be associated with HSPA1A induction.

Soybean and tempeh also showed neuroprotective effects in animal experiments. Using normal rat memory and learning abilities, Hamad et al. [100] examined the neuroprotective potential of soybean and tempeh extracts. The administration of soybean and tempeh extracts improved memory, although tempeh improved more than soybean (*p* < 0.05). Furthermore, tempeh extract exhibited a significant increase in ACh level and a greater decrease in inflammation than soybean extract. Chan et al. [101] examined tempeh’s cognitive and oxidative effects on SAMP8 mice. Increased catalase and SOD activity in the cortex, hippocampus, and striatum were observed after consuming tempeh. The highest concentration of tempeh treatment reduced Aβ and β-site amyloid precursor protein cleaving enzyme 1, increased catalase and SOD expression, and increased Nrf2 expression while lowering p-JNK and p-p38. The study found that tempeh can protect neurons from Aβ-induced damage and oxidative stress, and ameliorate memory impairment through modulating Nrf2 via the MAPK pathway [146,147]. Prediabetes can impair spatial memory and cognitive function by affecting the CNS. Ayuningtyas et al. [102] studied tempeh’s effect on spatial memory in prediabetic rats. The tempeh improved spatial memory in prediabetic mice by decreasing travel time across the Morris water maze. These studies show that tempeh can help people with dementia, AD, and prediabetes by improving their cognitive function.

Doenjang, a traditional Korean fermented soybean paste, possesses neuroprotective properties. The effects of doenjang on neurodegeneration and neuroinflammation in the hippocampus and cortex of high-fat diet-fed mice were studied by Ko et al. [106]. Doenjang treatment increased neurotrophic factor mRNA levels and reduced hippocampal neuronal loss, neuroinflammation, oxidative stress, Aβ levels, and tau hyperphosphorylation. The study indicated that there may be an increase in bioactive compounds produced during the fermentation and aging of soybeans, which potentially enhance neuroprotective effects.

### 5.4. Fermented Plant Root Products

Several plant roots and tubers are fermented to provide nutrient-dense foods. Many plant roots are being researched for their neuroprotective properties, and some have shown potential for reducing AD symptoms.

Codonopsis lanceolate is a traditional Asian herb. Codonopsis lanceolate root contains alkaloids, polyphenols, saponins, tannins, steroids, and triterpenes. These bioactive compounds treat illnesses including cough, bronchitis, spasm, inflammation, psychoneurosis, and cancer [148]. Codonopsis lanceolate fermentation revealed greater functional properties. He et al. [111] investigated the effects of fermented Codonopsis lanceolate extracts on cognitive, antioxidant, enzymatic, antibacterial, and cytotoxic properties. The results showed that fermented Codonopsis lanceolate extracts with probiotics increased phenol levels, particularly Cinnamic acid, and improved scopolamine-induced memory impairment in mice. According to Weon et al. [112], steamed and fermented Codonopsis lanceolate improved cognitive function in scopolamine-induced mice. Fermented Codonopsis lanceolate reduced memory impairment caused by scopolamine, inhibited AChE activity and increased BDNF level and CREB phosphorylation in hippocampal tissue.

Garlic (*Alium sativum* L.) possesses the antioxidant potential and improves spatial memory in rodents. Hermawati et al. [113] found that black garlic treatment in MSG-exposed rats improved spatial memory and increased hippocampus pyramidal cell numbers. Likewise, Nillert et al. [114] found that aged garlic extract (AGE) protects against neuroinflammation and cognitive impairment in Aβ-rats. The results showed that AGE improved short-term memory and reduced inflammation by suppressing microglial activation and IL-1β levels. According to Wichai et al. [115], AGE affects spatial memory and oxidative damage in Aβ-induced rats’ brains. It improved memory, reversed hippocampus neuronal loss, increased GPx and SOD activity, and reduced MDA levels in Aβ-induced neurotoxicity mice. These findings support AGE’s antioxidant potential in treating cognitive impairment.

Ginseng, or Panax ginseng Meyer, has been utilized in traditional herbal medicine for thousands of years. There are about 60 ginsenosides in ginseng root including Rb1-Rb3, Rc, Rd, Re, Rg1-Rg3, and other bioactive compounds such as polyacetylenic alcohols, polysaccharides, oligopeptides, and fatty acids, which has cognitive enhancing properties [149]. Lee and Oh [116] showed that the red ginseng treatment in aged mice increased Nrf2 and HO-1 antioxidative enzymes and attenuated iNOS, TNF-α, IL-1β, and COX-2 expressions. In addition, Tasi et al. [117] found that fermented Radix notoginseng, NB34, has a similar effect to Memantine in reducing APP and tau internal ribosome entry site activities. NB34 also improved spatial memory in ApoE-/- mice on a high-fat diet. In scopolamine-induced mice, Han et al. [118] studied the memory-enhancing effects of fermented wild ginseng root culture (HLJG0701). Spatial memory was improved and ACh and BDNF levels were increased in memory-deficient mice treated with HLJG0701. Similarly, Kim et al. [120] studied the neuroprotective and antioxidant properties of HLJG0701-β. In scopolamine-induced mice, treatment with HLJG0701 improved long-term memory, inhibited AChE activity, increased ACh and blood catalase levels, and reduced MDA and oxidative stress levels in the brain, indicating HLJG0701 is an effective cognitive enhancer.

### 5.5. Fermented Fruit and Vegetable Products

Fermenting fruits and vegetables had been used since ancient times to keep food fresh and retain nutrients. Fermentation has been shown to improve nutrient content in fruits and vegetables.

Papaya is the fruit of Carica papaya. It is native to the tropics of Central and Northern South America and is now grown in Malaysia, Indonesia, the Philippines, India, Australia, and Hawaii. Fermented papaya has been shown to have potent neuroprotective and antioxidant properties [150]. Imao et al. [121] reported that yeast fermented Carica papaya Linn (PS-501) in scopolamine-induced mice significantly improved memory and learning. Moreover, Yoshino et al. [122] investigated the antioxidant properties of fermented papaya preparation (FPP) in spontaneously hypertensive rats. The study reported an increase in antioxidant defenses in the spontaneously hypertensive rat brain, which may be attributed to the administration of FPP.

Jujube is the fruit of *Ziziphus jujuba* Mill (Rhamnaceae family). It has been used medicinally throughout Asia for centuries. Jujube includes neuroprotective flavonoids, polysaccharides, saponins, alkaloids, and triterpenoids [151,152,153]. Kim et al. [123] examined the neuroprotective effects of Zizyphus jujuba (Zj) and Zizyphus jujuba fermented by yeast (Zj-Y) on Aβ25-35-induced cognitive deficit AD mice. Zj and Zj-Y treatment in Aβ25-35-induced mice improved cognitive deficit and suppressed the elevations of NO and MDA in the brain, liver, and kidney. However, Zj-Y had greater scavenging effects against NO and MDA than Zj. The study found Zj-Y to be more effective than Zj in mitigating oxidative stress.

Kimchi is a fermented Korean cuisine. It is made by the fermentation of Chinese cabbage and radish with the addition of seasonings, including red pepper powder, ginger, garlic, green onion, and salts. Spontaneous fermentation of kimchi produces lactic acid bacteria including *Lactobacillus* sp. and *Leuconostoc* sp., which contributes to numerous health benefits, including neuroprotection [154]. Jung et al. [124] found that *Lactobacillus pentosus* var. *plantarum* C29 isolated from kimchi improved scopolamine-induced memory impairment in Morris water maze and Y-maze tests by inducing hippocampal p-CREB and BDNF expressions. Woo et al. [125] investigated the kimchi bioactive component’s ability to prevent endoplasmic reticulum (ER) stress-induced apoptosis in Aβ-mice. The bioactive compounds such as quercetin, KME, and HDMPPA significantly decreased oxidative stress, ER stress markers expressions (GRP78, p-eIF2α, p-PERK, XBP1, and CHOP), and pro-apoptotic molecules (p-JNK, Bax, cleaved caspases-3 and -9). Further investigation by Woo et al. [126] in Aβ25-35-induced cognitive deficit mice, kimchi improved cognition, increased GSH levels, and reduced TBARS, peroxynitrite, and ROS levels. Kimchi is also reported to enhance antioxidant enzyme levels and reduce inflammation-related enzymes, suggesting its potency in attenuating AD symptoms.

Moreover, Hong et al. [127] studied the effects of highbush blueberry (*Vaccinium corymbosum* L.) vinegar (BV) on cognitively impaired mice. BV administration significantly ameliorated cognitive impairment, inhibited AChE activity, facilitated cholinergic activity, and rehabilitated hippocampal cornu ammonis 1 neurons. The findings reveal that BV improved memory through activating the hippocampus BDNF/CREB/AKT signaling pathway.

### 5.6. Other Fermented Plant Products

*Rhus verniciflua*, the Chinese lacquer tree, is native to Asia. *Rhus verniciflua* is an antioxidant, anti-inflammatory, and anti-cancer agent. However, due to urushiol, an allergen, its medical usage was severely neglected. There are several ways to extract urushiol from *Rhus verniciflua*. One method involves fermenting mushrooms to lower urushiol concentration in *Rhus verniciflua*. Byun et al. [128] reported that the administration of RVH-1 or RVH-2 (constituents isolated from the mushroom-fermented stem bark of *Rhus verniciflua*) in excitotoxic mice decreased pyramidal neuronal cell death and hippocampal microglia activation. The study reveals that detoxified *Rhus verniciflua* has neuroprotective properties against kainic acid-induced excitotoxicity and thus has potential in herbal medicine.

Fermented teas such as black and Oolong tea have been shown to be neuroprotective. Mathiyazhagan et al. [129] investigated the effects of black tea extract (BTE) in cognitive deficit rats. BTE improved memory and AChE activity, and reduced oxidative stress and Aβ1–42 associated protein expressions. The study suggests that the neuroprotective properties of BTE may be due to the synergistic action of polyphenols found in black tea. Likewise, Cai et al. [130] reported that CDT-1 and CDT-2 from Chinese dark tea attenuated ubiquitinated protein aggregates and Aβ metabolic pathway, downregulated 4-HNE formation, enhanced endogenous antioxidant capacity, and protected neurons by reducing oxidative stress-aggregates cycle in SAMP8 mice.

### 5.7. Fungi

Medicinal fungi have proven antioxidant and anti-inflammation properties in traditional Chinese medicine. The effects of fermented medicinal fungus on cognitive function have recently been studied in animal models.

*Ganoderma lucidum* is a prominent medicinal mushroom in Asia. Phytochemicals in *Ganoderma lucidum*, such as proteins and steroids, may contribute to its antioxidant, anti-inflammatory, and neuroprotective properties, as well as improving memory and immunity [155,156]. Choi et al. [131] investigated the anti-amnesic efficacy of *Ganoderma lucidum* water extracts by two-step fermentation with lactic acid bacterium *Bifidobacterium bifidum* and *Lactobacillus sakei* LI033 on scopolamine-induced rats. The study showed that the secondary fermented extracts significantly reduced the escape latencies in the Morris water maze test, increased swimming times within the target zone during the probe trial, and lowered AChE activities in the brain, revealing the significance of secondary fermentation with lactic acid bacteria in enhancing memory and cognitive functions.

*Cordyceps sinensis (Berk) Sacc.* is an entomogenous fungus used in traditional Chinese medicine to treat illnesses. *Cordyceps sinensis* and its derivatives have shown potential neuroprotective effects against acute brain injury [157]. Chen et al. [132] studied the therapeutic effects of Cs-C-Q80 (a pleiotropic Cordyceps sinensis analog) in vascular dementia mice. The study found that Cs-C-Q80 ameliorated memory impairment, decreased MBP expression and white matter rarefaction in the corpus callosum, and inhibited astrocytes activation and IL-1β and TNF-α expressions, indicating the anti-inflammatory properties of Cs-C-Q80.

*Cordyceps cicadae* is a Chinese edible mushroom. It contains antioxidant, anti-inflammatory, anti-aging, and neuroprotective components such as polysaccharide, adenosine, and N(6)-(2-Hydroxyethyl) adenosine [158]. Recently, Wu and Lee [133] reported that *Cordyceps cicadae* NTTU 868 improved memory deficit and decreased pro-inflammatory cytokine expressions and Aβ40 accumulation in streptozotocin-induced rat.

## 6. Preclinical In Vitro Studies

Table 4 lists the most recent and highly cited preclinical in vitro studies on fermented food extracts and their neuroprotective effects on brain cells.

The neuroprotective efficacy of kefir has been well established in both clinical and preclinical in vivo studies. Nevertheless, a recent in vitro study revealed that the pre-treatment with kefir water for 48 h enhanced the endogenous antioxidant levels in hydrogen peroxide (H_2_O_2_)-treated oxidatively stressed SH-SY5Y cells [159]. The study showed that kefir water B treatment which exhibited high TPC, TFC, FRAP, and DPPH activities resulted in greater cytoprotection in MTT assay, significantly lower percentage of necrotic cells in PI/Annexin V-FITC assay, greater protection to cytoplasmic and cytoskeleton inclusion of SH-SY5Y cells, upregulation of SOD and catalase activities, and downregulation of Tp73, signifying its ability in conserving neurons from degeneration due to oxidative stress. Interestingly, Lee et al. [160] assessed the neuroprotective activity of lactic acid bacteria fermented mango peel on Aβ-induced Neuron-2A cell damage. The study showed that the *Lactobacillus acidophilus* (BCRC14079) fermented mango peel extracts upregulated BDNF expressions, attenuated oxidative stress, reduced Aβ accumulation, and decreased the elevation of subG1 caused by Aβ induction in Neuron-2A cells. In addition, Lee et al. [161] reported the neuroprotective effects of *Leuconostoc mesenteroides* H40 isolated from kimchi in H_2_O_2_-treated SH-SY5Y cells. The study showed that treatment with *Leuconostoc mesenteroides* H40 confirmed an increase in cell viability, significantly enhanced BDNF expression, and reduced Bax/Bcl-2 ratio in H_2_O_2_-induced SH-SY5Y cells, indicating its use as prophylactic functional dairy food. Likewise, Cheon et al. [162] also reported the neuroprotective effects of heat-killed *Lactobacillus fermentum* KU200060, *Lactobacillus delbrueckii* KU200171, and *Lactobacillus buchneri* KU200793 isolated from kimchi on 1-methyl-4-phenylpyridinium (MPP+)-induced cytotoxicity in SH-SY5Y cells. The treatment with a conditioned medium containing heat-killed *Lactobacillus buchneri* KU200793 resulted in the highest levels of BDNF and a significant decrease in the Bax/Bcl-2 ratio in SH-SY5Y cells treated with MPP+. Having great neuroprotective and probiotic potentials, the study suggests that *Lactobacillus buchneri* KU200793 can be used as a prophylactic functional food source.

*Cornus officinalis*, a Japanese cornelian cherry, belongs to the family of Cornaceae. It is a traditional medicine with strong anti-inflammatory [170] and antioxidant [171] properties as it contains a large number of phenolic and flavonoid compounds. Tian et al. [163] compared the neuroprotective effects of *Lactobacillus rhamnosus*, *Enterococcus faecium*, and *Lactobacillus acidophilus* fermented *Cornus officinalis* (FCC) with original *Cornus officinalis* (CC) on H_2_O_2_-induced SH-SY5Y cells. The study showed that both CC and FCC significantly inhibited ROS generation, and lactate dehydrogenase release, and significantly enhanced the antioxidant and neuronal marker expressions, such as catalase, SOD, and BDNF. In addition, the CC and FCC pre-treatment regulated the Bax/Bcl-2 ratio and downregulated MAPK phosphorylation. The study demonstrated that CC and especially FCC attenuated oxidative stress by blocking the MAPK signaling pathway and thus, may be used as a potential neuroprotective agent.

Moreover, Zhang et al. [164] studied the neurite outgrowth-promoting properties and neuroprotective effects of oolong tea against glutamate-induced death in Neuro-2A and HT22 cells. Treatment with the oolong tea extracts resulted in decreased intracellular ROS accumulation and increased cellular antioxidant enzyme activities such as SODs, GPx, and GSTs. Treatment with oolong tea extracts also enhanced the average neurite length and the mRNA expression of GAP-43 and Ten-4 in Neuro-2A cells. The results indicated that the oolong tea extracts could be a potential candidate for preventing neurodegenerative diseases. Similarly, Rho et al. [165] studied the inhibitory activities of fermented tea, *Camellia sinensis,* against Aβ-induced cytotoxicity in SH-SY5Y cells. The study reported that among the thirty-three identified compounds, three tea polyphenols such as (–)-catechin gallate (CG), (–)-epicatechin gallate (ECG), and (–)-epigallocatechin gallate (EGCG) significantly reduced Aβ aggregation in ThT fluorescence-based assay. This anti-Aβ aggregation effect of these three compounds was consistent with transmission electron microscopy analysis. In addition, the study found that among the three compounds, CG and ECG demonstrated stronger protection against Aβ-induced toxicity in SH-SY5Y cells, particularly CG. The study indicated that the CG remarkably showed more potent inhibitory activity than EGCG, the best anti-Aβ aggregation agent from the fermented tea.

Tempeh revealed significant neuroprotective and memory and cognition ameliorating effects on both clinical and preclinical in vivo studies. Hwang et al. [43] recently reported the promising in vitro neuroprotective effects of tempeh against lipopolysaccharide (LPS)-induced damage in BV2 microglial cells. The study showed that red beans fermented through co-cultivation with *Rhizopus* and *Lactobacillus* (RT-C) having the highest γ-aminobutyric acid (GABA) and anthocyanin concentrations more effectively attenuated the generation of LPS-induced ROS, upregulated the expressions of BDNF, and downregulated the expressions of CREB and nitric oxide synthase in LPS-induced BV-2 cells. Studies have reported the important role of GABA in modulating inflammatory responses. GABA mitigates the systemic inflammatory cytokine productions, suppresses LPS, and induces a microglial response by inhibiting the inflammatory pathways mediated by p38 MAP kinase and NF-κB [172,173]. A highly cited study by Cho et al. [166] demonstrated the neuroprotective effects of GABA produced by *Lactobacillus buchneri* isolated from kimchi on H_2_O_2_-induced PC12 cells. Pre-treatment with GABA-producing *Lactobacillus buchneri* increased PC12 cell viability and showed complete neuroprotective by retaining 100% cell viability upon induction with H_2_O_2_. These studies indicate that GABA could be of great interest as a neuroprotective agent.

Furthermore, Yim et al. [167] assessed the enhancement of neuroprotective effects of different *Latobacillus* strains fermented Sagunja-tang in H_2_O_2_-induced SH-SY5Y cells. The study found that Sagunja-tang fermented with *Latobacillus plantarum* 166 treatment resulted in high protection against cell death induced by H_2_O_2_ and etoposide, suppressed ROS generation, and reduced mitochondrial membrane potential disruption in SH-SY5Y cells. The study suggests that the enhancement of neuroprotective effects might be due to the conversion of Sagunja-tang phytochemicals by fermentation, which can be a potential treatment option for neurological damage-related diseases. Musa et al. [168] also reported the neuroprotective effects of *Lactobacillus fermentum* LAB9 or *Lactobacillus casei* LABPC fermented cow’s milk (CM) on LPS-induced BV2 cells. Treatment with CM-LAB9 and CM-LABPC on LPS-induced cells showed a significant decrease in NO level without affecting cell viability, and there were no changes in the CD40 expression.

*Curcuma longa* L., commonly known as turmeric, is a well-known medicinal plant with numerous health benefits, including anti-inflammation, anti-cancer, and neuroprotective properties. However, studies on fermented *Curcuma longa* L. (FCL) and its neuroprotective activities are very limited. Eun et al. [169] studied the neuroprotective potential of FCL with *Lactobacillus plantarum* sp. on proinflammatory-activated murine BV2 microglial cells and oxidative stress-induced cell death in rat glioma C6 cells. The study showed that the FCL pre-treatment in C6 cells effectively prevented cell death induced by oxidative stressors such as tert-butyl hydroperoxide and H_2_O_2_. FCL also inhibited PGE2 and NO production by inhibiting COX-2 and iNOS expressions in BV2 cells in a dose-dependent manner. The inflammation and oxidative stress attenuating potentials of FCL can be a promising dietary agent in preventing and treating microglial-cell mediated neurodegenerative diseases.

## 7. Gut–Brain Axis

Intestinal microbial flora in healthy individuals plays essential physiological functions. They help digest food ingredients that are indigestible to give energy and nutrients to the host and maintain the immune system’s balance [174]. Over the years, studies have found a complex interaction between the host and its microorganisms, which affects brain function and behavior, in addition to digestion and energy utilization [175,176]. In fact, intestinal cells and these microorganisms have formed a symbiotic relationship by evolving together for a very long time. Now, some have even recognized this microbiome as an organ of the human body. In the complete human microbiome, the intestinal microbiome has a significant part in it. It plays a crucial role by interacting with various organ systems bi-directionally, especially with major parts of the central nervous system (CNS) through direct and indirect pathways [177]. This relationship between the intestinal microbiome and the CNS is often called the gut–brain axis.

**Figure 3:**
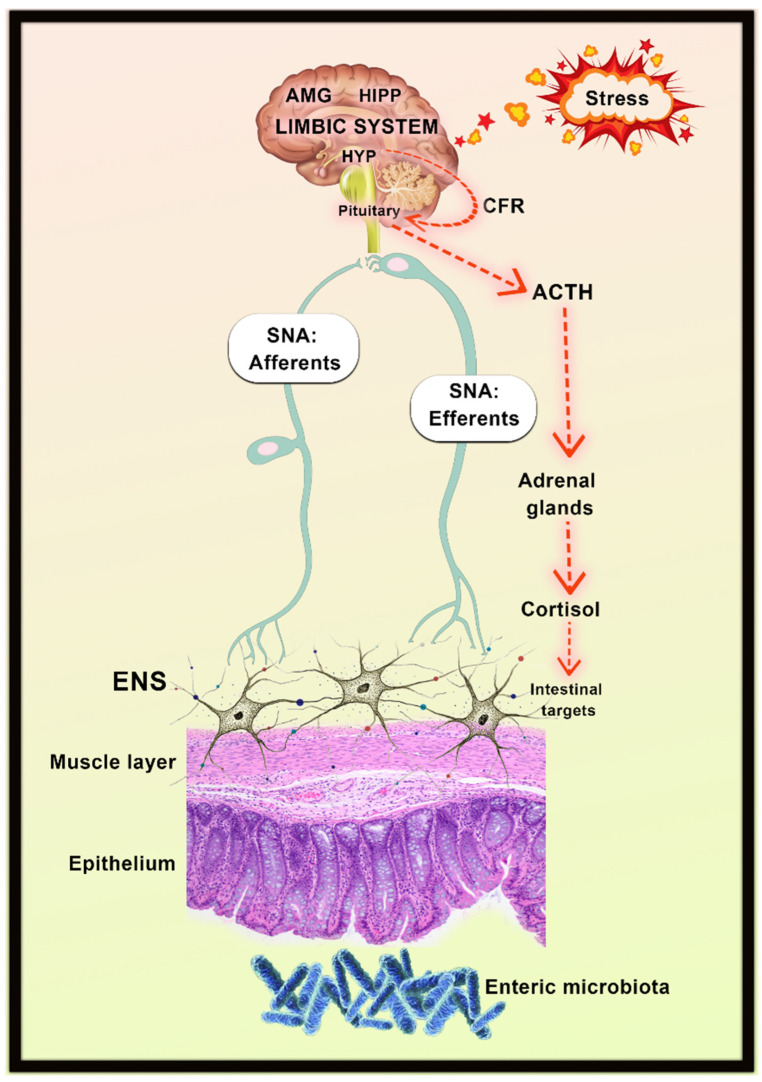
The structure of the microbiome–gut–brain axis. The HPA axis (in dashed line) in the CNS can be activated in response to environmental factors, including stress or emotion. Through a complex interaction between the AMG, HIPP, and HYP, constituting the limbic system, HPA is finalized to cortisol release. The secretion of the CRF from HYP stimulates the secretion of ACTH from the pituitary gland, leading to the release of cortisol from the adrenal glands. CNS communicates with afferent and efferent pathways with intestinal targets such as muscle layers and gut mucosa, ENS, modulating motility, permeability, immunity, and mucus secretion. The enteric microbiota has bidirectional communication with these intestinal targets that modulate the gastrointestinal functions and modulates itself by gut–brain interactions. HPA, hypothalamic pituitary adrenal; CNS, central nervous system; AMG, amygdala; HIPP, hippocampus; HYP, hypothalamus; CRF, corticotropin-releasing factor; ACTH, adrenocorticotropic hormone; ENS, enteric nervous system.

The bidirectional communication network of the gut–brain axis consists of the CNS (brain and spinal cord), the endocrine system (hypothalamic-pituitary-adrenal (HPA) axis), the enteric nervous system, the immune system (cytokines and chemokines), the autonomic nervous system (including efferent and afferent neurons), and the digestive system (Figure 3) [136,178]. It is mainly through the signaling of endocrine, neural, immune, and humoral from the gut microbiota to the brain and from the brain to gut microbiota. In addition, it is involved in the secretion of hormones, gut motility, and the production of bicarbonates, acid, and mucus [136]. Recent studies have demonstrated the notion of the gut–brain axis in germ-free rodents, probiotics, antibiotics, stool microbial transplantation, and gastrointestinal tract infections [177,179,180,181,182].

The autonomic system drives efferent and afferent signals from the CNS to the intestinal wall, rising from the lumen and transmitting through enteric, spinal, and vagal pathways to CNS, respectively [178]. The HPA axis plays a vital role in the stress response that coordinates the adaptive responses and comprises the interaction between the hypothalamus, pituitary gland, and adrenal glands [183]. It is a part of the limbic system which predominantly involved in memory and emotional responses. This system gets activated due to the environmental stress and the enhanced release of systemic pro-inflammatory cytokines via secretion of corticotropin-releasing factor (CRF) from the hypothalamus, which mediates the secretion of adrenocorticotropic hormone (ACTH) from the pituitary gland and in turn leads to the release of cortisol from the adrenal glands [183]. Cortisol is the primary stress hormone affecting the brain and other human organs. Hence, the combined interactions of both hormonal and neural lines allow the brain to influence the activities of intestinal functional effector cells such as epithelial cells, immune cells, smooth muscle cells, enteric neuronal cells, and interstitial cells enterochromaffin and Cajal cells [183]. Similarly, these cells are also influenced by the gut microbiota, which attributes to the communication roles in the gut–brain axis. The gut microbiota acts on the HPA axis and the vagus nerve to produce bacterial metabolites through tryptophan metabolism.

## 8. Gut Microbiota and Development of Alzheimer’s Disease

Recent experimental and clinical data shows the important role of gut dysbiosis and microbiota–host interactions in AD. Changes in the gut microbiota composition can cause elevated permeability of the gut barrier and activation of immune cells leading to systemic inflammation, which successively results in the blood–brain barrier impairment and promote neuroinflammation, neural injury, neuronal loss, and eventually AD [184]. In addition to the important role of gut microbiota in developing brain function and changes in an individual’s behavior, gut microbiota forms a significant amount of bacterial amyloids. Amyloids can be secreted by several gut microbiota members, including *E. coli*, *Bacillus subtilis*, *Pseudomonas fluorescens*, *S. typhimurium*, *Staphylococcus aureus*, etc. [185]. The best-studied bacterial amyloid is curli produced by *E. coli* [186]. Microbial amyloids such as CsgA have different amino acid sequences than human Aβ1-42. However, they both have the same pathogen-associated molecular patterns (PAMPs). Hence, they can interact with the same TLR2, triggering pro-inflammatory cytokines and activating systemic inflammation leading to neuroinflammation [187,188,189]. Chen et al. [190] investigated the role of curli-producing *E. coli* in aged rats and transgenic *C. elegans*. Rats exposed to curli-producing *E. coli* enhanced the deposition of neuronal alpha-synuclein in both the gut and brain and increased astrogliosis and microgliosis than rats exposed to no curli-producing bacteria. Likewise, *C. elegans* exposed to curli-producing *E. coli* had increased alpha-synuclein aggregation [190]. Additionally, animals exposed to curli-producing bacteria had an increased expression of TLR2, TNF, and IL-6 in the brain [190].

Lipopolysaccharide (LPS) is a large molecule of lipid and polysaccharide. It can be found in the outer membrane of Gram-negative bacteria. LPS is a crucial mediator between gut microbiota dysbiosis and AD pathology. It can interact with CD14 and the TLR4-MD-2 complexes of immune cells and cause a potent immune response. The further interaction of TLR4 with TIRAP and MyD88 activates the pro-inflammatory transcription factor, NF-κB. NF-κB promotes pro-inflammatory cytokine secretion and triggers pathogenic pathways in AD [191,192]. A study by Zhang et al. [193] showed increased plasma LPS levels in AD patients, which was positively associated with the activation of blood monocyte/macrophage levels. Another study by Zhan et al. [194] reported the colocalization of LPS with Aβ1-40/42 in amyloid plaques and around the vessels. Similarly, Zhao et al. [195] detected LPS in the superior temporal lobe neocortex and hippocampus in AD patients. A study using an animal model has shown that the LPS injection into the fourth ventricle of the brain reproduces many inflammatory and pathological features seen in AD [196]. Likewise, the LPS injection into the peritoneal cavity of mice resulted in a prolonged increase in Aβ in the hippocampus leading to cognitive deficits, indicating the role of LPS in amyloid fibrillogenesis [197,198]. Thus, gut inflammation might be a cause of AD pathogenesis.

Moreover, several studies reported the presence of pathogens in the post-mortem brains of patients with AD [195,199,200,201]. The reported pathogens are herpes simplex virus type 1 and bacteria such as *Borrelia burgdorferi*, *Chlamydophila pneumoniae*, or other *spirochetes* [202,203,204]. In addtion, Kountouras et al. [205] reported a significantly enhanced level of Helicobacter pylori-specific IgG antibodies in the AD patient’s cerebrospinal fluid and serum. Doulberis et al. [206] proved the hypothesis that Helicobacter pylori infection might influence the course of AD pleiotropically. According to the study, probable causes include Helicobacter pylori entering the brain via the oral-nasal-olfactory pathway or circulating monocytes infected with Helicobacter pylori undergoing faulty autophagy due to a disturbed blood–brain barrier, potentially triggering neurodegeneration [206]. Hence, modulation of the gut microbiota with probiotic supplementation or fermented foods rich in probiotics may create new preventive and therapeutic options in AD.

## 9. Potential Mechanism of Fermented Foods on Gut–Brain Axis

### 9.1. Chemical Constituents Modulation

Fermented foods contain various microbes, which may help modulate the chemical constituents, improving the bioavailability and the activity of the fermented foods. A study showed the chemical changes during the fermentation process, such as increasing bioactive peptides and creating phytochemicals [207]. The components obtained by incubating the natural foods with microbes enhance their neuroprotective effects by increasing their bioavailability through intestinal absorption and utilization of the ingested nutrients within the body [207,208]. The intestinal epithelium is a selectively permeable barrier that prevents access to harmful substances. Therefore, the absorption of the nutritional components is restricted in the intestines, and sometimes, these nutritional components are required to be converted into an active form by the intestinal microbes [209]. As reported by a recent study, short-chain fatty acids (SCFA), which are intestinal bacteria’s fermented products, positively influence the host metabolism and are shown to have an essential function in the CNS [210]. Previous studies also showed that resident intestinal microbiota could control phytochemical absorption and its anti-inflammatory and antioxidant functions [211,212,213]. Another study by Hooper et al. [209] has demonstrated that microbial colonization on germ-free mice results in significant changes in the transcription of genes associated with nutrient absorption and metabolism. Thus, the studies suggest that the proper fermentation of foods could increase their beneficial contents in promoting biological function and bioavailability.

### 9.2. HPA Axis Inhibition

Recent studies have reported that gut microbiota plays a crucial function in the CNS, which results in altered brain function and behavior [176,181,214,215]. Furthermore, it was found that the gut microbiota is significantly associated with the HPA axis. A study by Sudo et al. [216] showed that in germ-free mice, moderated levels of stress-induced HPA reactivity with excessive secretion of corticosterone and adrenocorticotrophin were compared to the specific pathogen-free controls. Studies also showed that the administration of probiotics attenuates the HPA-induced stress responses [217,218].

Xu et al. [219] studied the preventive effects of *Lactobacillus rhamnosus* (*L. rhamnosus*) zz-1 against depression on a mouse model with chronic unpredictable mild stress (CUMS). The study showed that the administration of *L. rhamnosus* zz-1 ameliorated CUMS-induced depression-like behavior of mice with decreased body growth rate, increased immobility time, lowered sucrose preference, and reduced curiosity and mobility. Additionally, treatment with *L. rhamnosus* zz-1 significantly inhibited hormone release caused by hyperactivity of the HPA axis, relieving CUMS-induced monoamine neurotransmitter deficiencies while also increasing BDNF and TrkB expression [219]. Treatment with *L. rhamnosus* zz-1 was also reported to reduce intestinal inflammation and alleviate intestinal damage in depressed mice [219]. Meanwhile, *L. rhamnosus* zz-1 significantly restored the dysbiosis of the mouse gut microbiota caused by CUMS, such as alterations in the abundance of *Lachnospiraceae* NK4A136, *Bacteroides*, and *Muribaculum* [219]. These findings showed that *L. rhamnosus* zz-1 was beneficial in reducing depression caused by chronic stress, adding to the body of evidence supporting probiotics’ mental health benefits.

Since the HPA axis hyperactivity is associated with cognitive impairments, it is suggested that the consumption of fermented foods rich in probiotics might improve cognitive functions by normalizing the HPA activity.

### 9.3. Neurochemical Modulation

Another potential mechanism of fermented foods that can influence the brain is neurochemical modulation. The secretory epithelial cells in the gut microbiota secrete many neurometabolites by the stimulatory action of the microbiota [136]. These neurometabolites consist of neurotransmitters that directly act on the CNS signaling cascades and other biochemical effectors that directly or indirectly affect CNS health [220,221]. Bacterial strains, such as *Lactobacillus* and *Bifidobacterium*, can produce high quantities of GABA in the presence of a suitable substrate [222]. The communication of neurometabolites from the gut to the CNS is through the vagal afferent’s stimulation and via their distal endocrine action after being absorbed into the bloodstream [220]. Any alteration in neurotransmitter levels leads to behavioral changes, including elevated spontaneous motor activity from enhanced dopamine levels, noradrenaline, and serotonin in the striatum [220]. Neurochemical modulation is critical in neurodegenerative disease management as there is often a dysregulation in the production of neurotransmitters in these conditions that eventually promote disease progression.

Several previous studies have revealed that gut microbiota affects the neurotransmitter systems. Studies on germ-free mice and mice infected with dysbiosis have shown decreased hippocampal BDNF expressions, which are crucial for the survival and differentiation of neurons [216,223,224]. The studies showed that probiotic supplementation reverted the expression of BDNF to the control level. Variation in the level of GABA, which is a primary inhibitory neurotransmitter, was also observed. The expression of specific GABA receptors was reformed in a regional-dependent manner upon chronic administration of probiotic *Lactobacillus rhamnosus*. The study indicated that the treatment of *Lactobacillus rhamnosus* leads to a key route to gut–brain communication [225]. Barrett et al. [226] have reported that some probiotic bacteria are able to produce GABA from glutamate in culture. In addition, some other experiments have demonstrated that the probiotic administration changes the serotonin turnover and its related metabolites in the brain [227,228]. Neurotransmitters such as glutamate, serotonin, BDNF, and GABA are shown to be respectively involved in memory and learning [229,230]. Hence, fermented foods can improve cognitive functions by modulating the secretion of neurotransmitters.

## 10. Microbiota Modulation as a Therapeutic Target in Alzheimer’s Disease

Numerous studies proved the beneficial effects of probiotics by reducing pro-inflammatory response, protecting against barrier disruption, increasing intestinal epithelial integrity, and inhibiting the activation of neuroinflammation and neurodegeneration [231,232]. For instance, an in vitro study showed that *Lactobacillus rhamnosus* and *Enterococcus faecium* decreased the production of TNF-α [233]. Additionally, in animal studies, the administration of these probiotic strains induced antioxidant enzymes and reduced oxidative stress markers in the brain [233]. Several other studies using animals support the therapeutic potential of Lactobacilli and Bifidobacteria [77,168,234,235]. Musa et al. [168] suggested that the attenuation of LPS-induced memory deficit and neuroinflammation by probiotics might be mediated via anti-inflammation by inhibiting AChE and antioxidant activities. Similarly, in a clinical trial, Akbari et al. [77] showed supplementation with Lactobacilli, and Bifidobacteria-based probiotics significantly enhanced the Mini-Mental State Examination scores in AD patients.

In addition, fecal microbiota transplantation has also been used in many animal models exploring its therapeutic effects on neurodegenerative disorders. A study by Sun et al. [236] found that the fecal microbiota transplant in APPswe/PS1dE9 transgenic mouse model improved cognitive deficits, reduced the brain deposition of Aβ, decreased phosphorylation of tau protein and the levels of Aβ40 and Aβ42, increased synaptic plasticity, and decreased COX-2 and CD11b levels. A case study by Park et al. [237] showed that fecal microbiota transplant resulted in improved cognitive function, changed microbiota composition in recipient feces, and showed significantly different short-chain fatty acids. The study indicates the presence of an association between the gut microbiome and cognitive function. However, reports on the therapeutic potential of fecal microbiota transplant on AD are minimal.

Another option for gut microbiota modulation is antibiotic treatment. Antibiotics can treat small intestinal bacterial overgrowth (SIBO) and intestinal colonization by pathogenic strains. Interestingly, treatment of SIBO with rifaximin in PD patients improved gastrointestinal symptoms and motor fluctuations [238].

However, dietary intervention is the most effective approach to modifying the gut microbiota. Food-based therapies are shown to influence the gut microbial composition or directly affect neural functioning in both the ENS and CNS [239,240]. A healthy diet consisting of plant-based foods, probiotics, antioxidants, omega-3 polyunsaturated fatty acids, soybeans, and nuts has been shown to attenuate neuroinflammatory response and decrease the risk of cognitive deficit, and eventually AD [241,242].

## 11. Commercialized Fermented Products

Consumers are now aware that food fermentation can improve nutrient content, organoleptic characteristics, and health. Thousands of commercially fermented foods have invaded the market. For example, in 2018, roughly 64.9 million tonnes of milk were fermented into cheese and other fermented goods [6]. According to the latest estimates, the global fermented foods and ingredients market was valued at USD 565.09 billion in 2019 and is expected to reach USD 875.21 billion by 2027 [243].

Food fermentation has progressed from traditionally fermented meals to tailored fermented foods for health-conscious and lactose-intolerant consumers. The WHO defines probiotics as “live microorganisms which when administered in adequate amounts confer a health benefit on the host”. Several research have documented the probiotic effects of fermented foods [7,9,29,30,33,39,40,72,244,245]. Today’s most widely accessible probiotics are *Lactobacillus* and *Bifidobacterium* species. Minoru Shirota, who produced the popular probiotic product Yakult, discovered *Lactobacillus casei* strain Shirota in the 1930s. A German company has also marketed salami containing three intestinal LAB (*Lactobacillus acidophilus*, *Lactobacillus casei*, and *Bifidobacterium* spp.) as a probiotic [182]. Soon after, a Japanese business launched a probiotic fermented meat spread (*Lactobacillus rhamnosus* FERM P-15120). Various commercial probiotic products produced from marine fermentation techniques were presented by Hayes and Garca-Vaquero [245]. This includes Prolastin (hydrolyzed fish skin) from Copalis, France, and Pepha-Tight (fermented microalgae) from Pentapharm, Switzerland.

## 12. Future Prospects and Limitations

Multidisciplinary omics methods have been used for many years to detect microbes isolated from food fermentations. This method allows for the metabolic engineering of fermented foods and beverages. This may open up new avenues for creating innovative food fermentation applications with favourable features. The expanding health-conscious population is also driving demand for probiotics in fortified foods. As previously stated, *Lactobacillus* (mainly LAB) is the major genus in fermented products and has been authorized by the FDA as Generally Recognized as Safe (GRAS). Collectively, this may be a key to commercializing fermented products and achieving consumer pleasure. However, when it comes to marketing fermented products, the information available online (websites and social media) is sometimes incorrect or exaggerated. Thus, consumers may lose their right to accurate information regarding fermented foods’ nutritional or other special features. To classify a product as a probiotic fermented food, it must include health-promoting bacteria. Thus, the terms ‘fermented foods’ and ‘probiotics’ cannot be used interchangeably [23].

The value of fermented foods as a medicinal product is becoming clearer as probiotic research advances. Synthetic supplements have been linked to harmful consequences such as DNA damage, apoptosis, and carcinogenicity [246]. Thus, naturally fermented foods can be used as probiotic supplements instead of expensive probiotic formulations. Fermented foods also have a number of anti-aging advantages, including extending lifespan and improving overall health. Fermented soybeans, for example, have shown promise due to chemicals like genistein and daidzein [247]. However, consumers’ adoption of new items is an important factor to consider. Consumers prefer products that are appealing to them over products that are functional. Thus, greater research into their microbiological, physical, and chemical qualities to improve their sensory features should be undertaken. One of the main disadvantages of food fermentation is the excessive salt content. Salt is used in fermentation to desalt vegetables and fish before converting them into desired products. So, finding a better substitute for salt, or at least limiting its usage, is critical.

Notably, fermented foods may help combat malnutrition in many underdeveloped countries. Fermented foods provide more important amino acids, fatty acids, proteins, and vitamins [28]. So many fermented foods are made to combat global food insecurity. The manufacturing of fermented foods is also ascribed to their socio-economic relevance, providing millions of people with job opportunities and income. While fermentation is an ancient food process, only a few artisanal items have been scientifically tested. Consumption of fermented foods may boost nutrient absorption. However, many traditional household fermentation procedures are unsanitary, increasing the risk of disease outbreaks. Progress in industrial biotechnology should be the new norm to achieve the United Nations’ Sustainable Development Goals on hunger (SDG2).

## Figures and Tables

**Table 1:** Health-promoting compounds in fermented foods.

Health-Promoting Activity	Health-Promoting Compounds	Fermented Food Products	Fermenting Microorganism(s)	Reference
Neuroprotection	Antioxidant enzymes, GABA, genistein, anthocyanin	Sausage	*Enterococcus thailandicus*, *Enterococcus faecalis*	[41]
		Soybean	*Bacillus amyloliquefaciens*, *Bacillus amyloliquefaciens*, *Rhizopus oryzae*, *Pichia farinose*	[42]
		Tempeh	*Rhizopus* spp., *Lactobacillus* spp.	[43]
Anti-inflammatory	Polyphenol	Kefir	LAB	[44]
		Soy milk	*Lactococcus lactis* subsp. *lactis* S-SU2	[45]
Anti-hypertensive	ACE inhibitory peptides, GABA	Milk	*Lactobacillus helveticus* strain CP790, *Saccharomyces cerevisiae*	[46]
		Milk	*Lactobacillus casei* strain IMAU20411	[47]
		Goat milk	*Lactobacillus plantarum* strain 69	[48]
		Camel milk	*Lactobacillus rhamnosus* strain MTCC 5945 (NS4)	[49]
		Milk	*Lactococcus lactis* strain DIBCA2	[50]
		Milk	*Lactobacillus plantarum* strain PU11	[50]
		Soy milk	*Enterococcus faecium*	[51]
		Skim milk	*Lactobacillus plantarum*	[52]
		Milk	*Lactobacillus casei* strain Shirota, *Lactobacillus lactis* strain YIT 2027	[53]
		Cheese	*Lactobacillus lactis*	[54]
		Beans	*Bacillus subtilis* strain B060	[55]
Anti-cholesterol	Hydroxy-methylglutaric acid, Orotic acid (inhibitor of cholesterol synthesis)	Milk	*Lactobacillus acidophilus*	[56]
Anti-diabetic	Isoflavonoids, peptides	Soybeans (Meju)	*Bacillus* spp., *Aspergillus* spp.	[57]
		Cereal (Boza)	LAB	[58]
Anti-microbial	Bacteriocins,	Kefir Yoghurt	LAB	[59]
	Bacteriocin-like inhibitory substances (BLIS),	Ewe milk Yoghurt Buttermilk	LAB	[60]
	Carboxylic acids	Yak milk (Kurut)	LAB	[61]
		Cheese (Jben)	LAB	
		Wort	*Lactobacillus plantarum* strain FST1.7, *Lactobacillus brevis* strain R2D	
Anti-oxidative	Phenolic, flavonoid compounds	Wheat koji	*Aspergillus oryzae*, *Aspergillus awamori* strain nakazawa	[62]
		Cereal	Bacillus subtilis, Lactobacillus plantarum	[63]
		Wheat	*Aspergillus oryzae*, *Rhizopus oryzae*	[64]
		Wheat	*Aspergillus oryzae*, *Aspergillus niger*	[65]
		Soy whey	*Lactobacillus plantarum* strain B1-6	[66]
Anti-cancer	Peptides, Surfactin-like compounds	Soybeans (Cheonggukjang)	*Bacillus subtilis* strain CSY 191	[67]
		Camel milk	*Lactobacillus lactis*, *Lactobacillus acidophilus*	[68]
Alleviation of lactose intolerant	Lactase	Milk	*Lactobacillus acidophilus*	[69]
Anti-nutritive	Phytase	Bread	Yeast species	[70]

**Table 2:** Clinical studies of fermented foods and their effects on AD.

Fermented Food Products	Fermenting Microorganism(s)	Experimental Subjects	Assessments	Effects	Reference
Kefir fermented in milk	*Acetobacter aceti*, *Acetobacter* sp., *Lactobacillus delbrueckii delbrueckii*, *Lactobacillus fermentum*, *Lactobacillus fructivorans*, *Enterococcus faecium*, *Leuconostoc* spp., *Lactobacillus kefiranofaciens*, *Candida famata*, and *Candida krusei*	AD patients (*n* = 13)	Cognitive assessment, determination of cytokines, ROS, advanced oxidation protein products, MMP, p53, and cleaved PARP levels, cell cycle, cell viability, and apoptosis analyses	Marked improvement in memory, executive/language functions, and visual-spatial/abstraction abilities, decreased oxidative stress and inflammation, increased NO bioavailability, and improved serum protein oxidation, mitochondrial dysfunction, apoptosis, and DNA damage/repair.	[73]
Milk	*Lactobacillus helveticus* IDCC3801	Older people (60–75 years old)	Cognitive tests, PSS, BDNF, GDS-SF, and WBV	Improved cognitive function.	[74]
Milk	*Lactobacillus helveticus* CM4	Healthy adults (middle age)	RBANS test	Improved attention and delayed memory.	[75]
Soybean (DW2009)	*Lactobacillus plantarum* C29	Patients with mild cognitive impairment (*n* = 100) (55–85 years old)	Neurocognitive function tests, BDNF levels, and fecal microbiota analysis	Enhanced cognitive function, increased BDNF levels, and lactobacilli in the gut microbiota.	[76]
Probiotic milk	*Lactobacillus acidophilus*, *Lactobacillus casei*, *Bifidobacterium bifidum*, and *Lactobacillus fermentum*	AD patients (*n* = 60) (60–95 years old)	MMSE score and biomarkers test	Enhanced cognitive function and a significant decrease in MDA, hs-CRP, insulin metabolism markers, triglyceride, and VLDL.	[77]
Tofu, tempeh and other genistein-rich foods	Not reported as it is an observational study	Older people (*n* = 115) (52–98 years old)	Medical examination, cognitive and socioeconomic assessments	Improved memory and cognitive function in middle-aged people but not in older people.	[78]
Tempeh	*Rhizopus oligosporus*, Enterobacteriaceae and lactic acid bacteria	Older people with mild cognitive impairment (*n* = 90) (≥60 years old)	Cognitive tests and blood uric acid level	Improved global cognitive function	[79]
Soybean and soybean products	Not reported as it is a population study	Japanese subjects without dementia (60–79 years old)	Neuropsychological tests, dietary surveys, and health examinations	Reduced risk of dementia	[80]
Papaya	Yeast	Patients with initial or mild AD (*n* = 40) (mean age 78.2 ± 1.1 years)	Urinary 8-OHdG test	Significant decrease in the 8-OHdG levels	[81]

ROS, reactive oxygen species; MMP, mitochondrial membrane potential; p53, tumor protein; PARP, poly (ADP-ribose) polymerase; PSS, perceived stress scale; BDNF, brain-derived neurotrophic factor; GDS-SF, geriatric depression scale-short form; WBV, whole blood viscosity; RBANS, repeatable battery for the assessment of neuropsychological status; MMSE, mini-mental state examination; MDA, malondialdehyde; hs-CRP, high sensitivity C-reactive protein; VLDL, very-low-density lipoprotein; 8-OHdG, 8-hydroxy-2′-deoxyguanosine.

**Table 3:** Preclinical in vivo studies of fermented foods and their effects on AD.

Fermented Food Products	Fermenting Microorganism(s)	Experimental Subjects	Assessments	Effects	Reference
Dairy					
Camembert cheese	*Penicillium candidum*	C57BL/6J mice, CD-1 mice, and B6SJL-Tg mice (6–8 weeks old)	Aβ1–42 deposition analysis, anti-inflammatory and phagocytosis assays	Reduction of Aβ and inflammation increased BDNF and GDNF	[89]
Soymilk	*Lactobacillus plantarum* strain TWK10	Wistar rats (*n* = 30) (8 weeks old, 260–300 g)	Learning and memory, antihypertensive, biochemical and histological analysis	Significant decrease in blood pressure improved the learning ability and reduced the occurrence of dementia	[90]
Calpis sour milk whey	*Lactobacillus helveticus*	ddY mice (*n* = 255) (7 weeks old)	SABT and NORT	Significantly improved cognitive impairment and object recognition memory	[91]
Lactopeptides	Digested with enzyme from *Aspergillus melleus* and *Bacillus stearothermophilus*	C57BL/6J mice (7 and 22 months old) and Crl: CD1 (ICR) mice (6 weeks old)	SABT, NORT, and monoamine oxidase inhibitory and monoamine analyses	Improved memory function, inhibited monoamine oxidase-B activity, and enhanced dopamine levels in brain tissue	[92]
Tryptophan-related dipeptides	Digested by enzymes from *Aspergillus melleus*	C57BL/6J mice (newborn <7 day, 7 weeks, and 68-weeks old) and ICR mice (6 weeks old)	Electrophysiology, SABT, and NORT	Suppressed microglial inflammatory response, increased Aβ phagocytosis, improved cognitive and memory impairment	[93]
Tibetan fermented milk	Not reported	B6C3 mice (*n* = 12) (2 months old, 19.86 ± 3.37 g) APPswe/PS1dE9-transgenic mice (*n* = 36) (2 months old, 20.03 ± 3.52 g)	MWMT, NORT, immunohistochemistry, 16S rRNA sequencing, and taxonomic analysis of gut microbiota	Improved cognitive impairment, reduced Aβ deposition in the cerebral cortex and hippocampus, increased intestinal microbial diversity	[94]
β-lactolin, a whey-derived lacto-tetrapeptide	Not reported	B6SJL-Tg mice (2.5 months old) and B6; C3-Tg mice (3 months old)	Cytokine, synaptophysin, Aβ, and tau by ELISA, immunohistochemistry, dopamine analysis, NORT, and OFT	Ameliorated synaptophysin, dopamine, Aβ, BDNF, inflammatory cytokines, and IGF-1 levels, and improved impaired long-term object memory and behavioral abnormality	[95]
Legumes and Cereal					
Cheonggukjang	*Bacillus subtilis* MC31 and *Lactobacillus sakei* 383	ICR mice (*n* = 80) (6 weeks old)	PAT, NORT, AChE, MDA, SOD, and NGF detection, and histological analysis	Improved short- and long-term memory, NGF signaling pathway, NGF concentration, Bax/Bcl-2 levels, AChE and SOD activity	[96]
Cheonggukjang and soybeanss	*Bacillus licheniformis* SCD 111067P	Sprague Dawley rats (*n* = 80) (223 ± 16 g)	PAT, MWMT, and immunohistochemistry	Significantly reduced Aβ accumulation, ameliorated insulin signaling, improved cognitive functions, and glucose regulations	[97]
Red mould rice	*Monascus purpureus* NTU 568	Wistar rats (*n* = 49) (280–320 g)	PAT, MWMT, detection of TBARS, ROS, ApoE, β-secretase, sAPPα, and brain cholesterol levels in the hippocampusand cortex	Improved memory deficits, brain cholesterol level, oxidative stress and lipid peroxidation, decreased Aβ formation and deposition, and suppressed ApoE expression	[98]
Kurozu and Kurozu Moromi	Not reported	R1 mice (*n* =16) (10 weeks old) and P8 mice (*n* = 27) (12 weeks old)	MWMT, antioxidant assays, and detection of HSPA1A mRNA expression	Suppressed Aβ accumulation and cognitive dysfunction and enhanced HSPA1A mRNA expression	[99]
Soybean and Tempeh	*Rhizophus* sp.	Sprague Dawley rats (*n* = 96) (180 ± 20 g) (3–4 months old)	Radial arm maze, elevated plus maze, ACh and AChE assays, and IL-10 and IL-1β measurements	Tempeh showed significant improvement in memory, ACh and AChE activities, and a decrease in inflammation	[100]
Tempeh	*Rhizopusoligosporus* (BCR C 31750)	SMAP8 mice (*n* = 32) (6 months old) and SAMR1 mice (*n* = 18) (6 months old)	Cognitive evaluation, redox status analysis, and RT-PCR and western blot analyses of Nrf2, p-JNK, and p-p38 expressions	Stronger cognition, reduced Aβ, carbonyl protein, and MDA levels, enhanced Nrf2, catalase, and SOD activities	[101]
Tempeh	Not reported	Wistar rats (*n* = 15) (180–280 g) (2.5–3 months old)	MWMT	Improved spatial memory impairment	[102]
Defatted soybean powder	*Lactobacillus pentosus* var. *plantarum* C29	ICR mice (24–28 g) (6 weeks old)	PAT, Y-maze and MWMT, and detection of AChE and BDNF activity	Improved memory impairment, increased BDNF activity, and inhibited AChE activity	[103]
Soybean	*Lactobacillus plantarum* C29	B6SJL-Tg mice (4 months old)	Y-maze task, PAT, NORT, MWMT, pyrosequencing, and in vivo intestinal permeability assay	Improved cognitive function, significantly reduced Aβ, β/γ-secretases, NF-κB activation, and caspase-3 expression, and enhanced BDNF expression	[104]
Nanonutraceuticals of soybean	*Bacillus subtilis*	Wistar albino rats (180–200 g)	MWMT, PAT, and assays for AChE, MDA, protein carbonyl, and oxidative markers	Ameliorated learning and memory, AChE and antioxidant status, reduced MDA, protein carbonyl, and Aβ deposition	[105]
Doenjang	*Aspergillus oryzae* and *Bacillus licheniformis*	C57BL/6J mice (*n* = 47) (4 weeks old)	Brain tissue histopathology, MDA and protein carbonylation measurement, immunoblotting, and qPCR analyses	Enhanced neurotrophic factor mRNA levels, alleviated neuronal loss, reduced neuroinflammation- and oxidative stress-related mRNA levels and oxidative metabolites contents	[106]
Kefir					
Kefir and kefir fractions fermented in cow milk	*Lactobacillus kefiranofaciens*, *Lactobacillus kefiri*, *Acetobacter fabarum*, *Lactococcus lactis*, and Rickettsiales	*Drosophila melanogaster*	Total amyloid quantification, survival assay, rapid iterative negative geotaxis assay, and histopathological analysis	Improved climbing ability, vacuolar lesions, survival rate, and neurodegeneration index.	[107]
Probiotics Fermentation Technology (PFT) kefir grain product	*Lactobacillus kefiri* P-IF, *Lactobacillus kefiri* P-B1, *Kazachstania turicensis*, *Kazachstania unispora*, and *Kluyveromyces marxianus*	Albino mice (25–30 g)	NORT, MWMT, evaluation of Aβ1-42, ACh, MDA, Nrf2, NF-κB, TNF-α, and Caspase-3 levels	Attenuated neuronal degeneration improved cognition, restored ACh levels, reduced apoptosis, oxidative damage, and proinflammatory cytokine expression.	[108]
Kefir fermented in organic powdered milk	Not reported	Albino rats (*n* = 60) (150–200 g)	T-maze test, biochemical analysis, detection of cholesterol, TNF-α and IL-10 levels	Attenuated cognitive impairment, Aβ and tau pathology, lipid profile, oxidative stress, and Bax expression	[109]
Kefir fermented in milk	Not reported	Albino rats (*n* = 72) (200–250 g)	MWMT, estimation of brain tissue expression of MAPK, Tau protein, ACAT, CBS, Aβ42, MDA, and GSH, and histopathology	Improved memory, decreased MAPK, Tau, ACAT, CBS, Aβ42, MDA, and oxidative stress levels, and increased GSH levels	[110]
Plant Root					
Codonopsis lanceolata extract	*Bifidobacterium longum* and *Lactobacillus rhamnosus*	ICR mice (*n* = 40) (27.7 ± 2.4 g) (5 weeks old)	PAT	Improved memory deficit	[111]
Codonopsis lanceolata	*Bifidobacterium longum* (KACC 20587), *Lactobacillus acidophilus* (KACC 12419), and *Leuconostoc mesenteroides* (KACC 12312)	ICR mice (*n* = 35) (25–30 g) (3 weeks old)	MWMT, PAT, AChE, BDNF, and CREB level	Increased cognition, BDNF, and CREB expressions, and inhibited AChE activity.	[112]
Black garlic	No fermenting microorganism is involved	Wistar rats (*n* = 25) (3–4 weeks)	MWMT, and estimation of the total number of hippocampal pyramidal cells	Ameliorated memory deficits and estimated a higher total number of hippocampal pyramidal cells	[113]
Aged garlic	No fermenting microorganism is involved	Wistar rats (*n* = 48) (180–220 g)	NORT, immunohistochemistry, and western blotting analysis	Significant increase in short-term memory and decrease in inflammatory responses	[114]
Aged garlic	No fermenting microorganism is involved	Wistar rats (*n* = 48) (180–220 g) (8 weeks old)	MWMT, histological analysis, neurons quantification, and biochemical analysis	Improved learning and short-term memory impairment, reversed neuronal loss, and increased GSH and SOD activities	[115]
Red ginseng	Not reported	C57BL/6 mice (28–30 g) (21 months old)	Y-maze task, NORT, MWMT, and immunoblot analysis	Attenuated iNOS, TNF-α, IL-1β, and COX-2 expressions, restored GSH levels and increased Nrf2 and HO-1.	[116]
Radix notoginseng	*Lactobacillus* spp.	ApoE^−/−^ mice (*n* = 16) (10 weeks old)	MWMT	Ameliorated spatial memory	[117]
Wild ginseng root extract (HLJG0701)	Lactic acid bacteria	ICR mice (*n* = 48) (8 weeks old)	AChE, ACh and BDNF expressions, MWMT, and Y-maze test	Significant reduction in AChE activity, increased ACh and BDNF levels, improved memory	[118]
Ginseng	*Lactobacillus paracasei* A221	Wistar rats (300–350 g) (10 weeks old)	MWMT, immunofluorescence, and western blotting	Improved memory, caspase-3, and Iba-1 levels, and loss of hippocampal neurons	[119]
Wild ginseng root extract (HLJG0701-β)	*Pediococcus pentosaceus*	Male C57BL mice (18.37–23.92 g) (9 weeks old) and female C57BL mice (18.40–20.97 g) (9 weeks old)	MWMT, Y-maze task, measurement of AChE, ACh, MDA, and catalase levels	Ameliorated the long-term memory impairment, ACh, and catalase levels, and reduced AChE and MDA levels	[120]
Fruits and Vegetables					
Papaya	Yeast	Mice	SABT and PAT	Improved short- and long-term memory	[121]
Papaya	Yeast	SHR rat (350–450 g)	Electronspin resonance imaging analysis	Up-regulated the redox defense activity	[122]
Zizyphus jujuba	*Saccharomyces cerevisiae*	ICR mice (*n* = 28) (5 weeks old)	T-maze test, NORT, MWMT, and measurement of ALT, AST, MDA, and NO levels	Ameliorated cognitive function and suppressed the elevations of NO and MDA	[123]
Kimchi	No fermenting microorganism is involved	ICR mice (28–30 g)	PAT, Y-maze test, MWMT, and immunoblotting	Ameliorated memory impairment and increased BDNF and p-CREB expressions	[124]
Kimchi	No fermenting microorganism is involved	ICR mice (5 weeks old)	Measurement of ROS, TBARS, AD-related markers, endoplasmic reticulum stress markers, and apoptosis-related molecules	Reduced APP, p-Tau, BACE, endoplasmic reticulum stress markers, and pro-apoptotic molecules, and enhanced cIAP and Bcl-2 expressions	[125]
Kimchi	No fermenting microorganism is involved	ICR mice (5 weeks old)	MWMT, NORT, T-maze test, measurement of ROS, peroxynitrite, TBARS, and GSH levels, and western blot analysis	Improved cognitive deficits and GSH level, and reduced TBARS, peroxynitrite, and ROS levels	[126]
Highbush blueberry	*Saccharomyces cerevisiae* KCCM 34709 and *Acetobacter* sp. KCCM 40085	ICR mice (6 weeks old)	Y-maze test, PAT, detection of ACh, AChE, SOD, catalase, and MDA levels, and immunohistochemistry	Significantly ameliorated cognitive functions, inhibited AChE activity, and facilitated ACh activity	[127]
Other Plant Products					
*Rhus verniciflua*	Mushroom-mediated fermentation. No fermenting microorganism is involved	ICR mice (23–25 g)	Immunohistochemistry and In situ labeling of DNA fragmentation	Significantly attenuated pyramidal neuronal cell death and microglia activation	[128]
Black tea	Fully-fermented tea produced through oxidation. No fermenting microorganism is involved	Albino Wistar rats (*n* = 36) (200–225 g) (10–12 weeks old)	PAT, MWMT, estimation of AChE, TBARS, SOD, GPx, and GSH levels, and western blot analysis	Improved memory deficits, inhibited AChE activity, reduced oxidative stress and Aβ1–42 related and apoptotic markers	[129]
Chinese dark tea	Not reported	SAMR1 mice (*n* = 8) and SAMP8 mice (*n* = 32) (25–30 g) (4 months old)	Measurement of oxidative stress- and Aβ42, H&E staining, Nissl dyeing, myelin staining, and Roche apoptotic staining	Attenuated Aβ metabolic pathway, downregulated 4-HNE formation, enhanced endogenous antioxidant capacity, and protected neurons by reducing oxidative stress	[130]
Fungi					
*Ganoderma lucidum*	*Bifidobacterium bifidum* and *Lactobacillus sakei* LI033	Sprague Dawley rats (*n* = 42) (200–250 g) (6 weeks old)	MWMT, PAT, rotarod test, vertical pole test, and measurement of AChE activity	Improved memory and lowered AChE activities in the brain	[131]
*Cordyceps sinensis (Berk) Sacc.*	Not reported	ICR mice (22–25 g) (8–10 weeks old)	NORT, MWMT, histopathology, immunohistochemistry, and western blot analysis	Improved learning and memory deficit, and significantly decreased MBP, TNF-α, and IL-1β expressions	[132]
*Cordyceps cicadae* NTTU 868	Fermented with potato dextrose broth powder and yeast extract	Sprague Dawley rats (*n* = 48) (6–8 weeks old)	MWMT and measurements of TNF-α, IL-1β, IL-6, and Aβ40-related proteins levels	Improved memory deficit, suppressed Aβ40, BACE, and pro-inflammatory cytokine expression, and increased MAGT1 expression	[133]

**Table 4:** Preclinical in vitro studies of fermented foods and their effects on AD.

Fermented Food Products	Fermenting Microorganism(s)	Experimental Subjects	Assessments	Effects	Reference
Kefir	Not reported	SH-SY5Y cells	Measurement of TPC, TFC, FRAP, and DPPH levels, MTT, AO/PI, Annexin V-FITC, SEM, TEM, and qPCR analysis for SOD, catalase, and Tp73 expressions	Increased TPC, TFC, FRAP, and DPPH activities, a significantly lower percentage of necrotic cells, greater protection to cytoplasmic and cytoskeleton inclusion of SH-SY5Y cells, upregulation of SOD and catalase activities, and downregulation of Tp73	[159]
Mango peel extracts	*Lactobacillus acidophilus* (BCRC14079)	Neuron-2A cells	MitoSOX-red stain, cell cycle, and immunocytochemistry	Upregulated BDNF expressions, attenuated oxidative stress, Aβ accumulation, and the elevation of subG1	[160]
Kimchi	*Leuconostoc mesenteroides* H40 *	SH-SY5Y cells	MTT assay and qPCR analysis of BDNF, Bax, and Bcl-2 expression	Increased cell viability and BDNF expression, and reduced Bax/Bcl-2 ratio	[161]
Kimchi	*Lactobacillus buchneri* KU200793 *	SH-SY5Y cells	MTT assay and qPCR analysis of BDNF, Bax, and Bcl-2 expression	Significantly increased BDNF expression and decreased Bax/Bcl-2 ratio	[162]
*Cornus officinalis*	*Lactobacillus rhamnosus*, *Enterococcus faecium*, and *Lactobacillus acidophilus*	SH-SY5Y cells	MTT assay, detection of ROS and LDH release, qPCR, and western blot analysis for Bax/Bcl-2 and MAPK expressions	Significantly inhibited ROS and LDH release, enhanced catalase, SOD, and BDNF expressions, and regulated the Bax/Bcl-2 ratio and MAPK phosphorylation.	[163]
Oolong tea	Semi-fermented Chinese tea produced through oxidation. No fermenting microorganism is involved	Neuro-2A and HT22 cells	MTT assay, measurement of ROS, and qRT-PCR analysis for SODs, GPx, and GSTs	Decreased ROS accumulation, increased SODs, GPx, GSTs, GAP-43, and Ten-4 expressions	[164]
*Camellia sinensis*	Fermented *Camellia sinensis* is produced via heating and enzymatic fermentation of leaves. No fermenting microorganism is involved	SH-SY5Y cells	ThT fluorescence-based assay, TEM, and CCK-8 assay	Significantly reduced Aβ aggregation and stronger protection against Aβ-induced toxicity	[165]
Tempeh	*Rhizopus* and *Lactobacillus*	BV2 cells	MTT assay, detection of ROS, and western immunoblot analysis for nitric oxide synthase, CREB, and BDNF expressions	Decreased ROS, CREB, and nitric oxide synthase levels, and upregulated BDNF expression	[43]
Kimchi	*Lactobacillus buchneri* *	PC12 cells	MTT assay	Increased cell viability and showed complete neuroprotection by retaining 100% cell viability	[166]
Sagunja-tang	*Lactobacillus rhamnosus* KFRI127, *Lactobacillus zeae* KFR129, *Lactobacillus rhamnosus* KFRI144, *Lactobacillus acidophilus* KFRI150, *Lactobacillus fermentum* KFRI162, *Lactobacillus plantarum* KFRI166, *Lactobacillus acidophilus* KFRI217, and *Lactobacillus helveticus* KFRI341	SH-SY5Y cells	CCK-8 assay, measurement of ROS, and MMPs assay	High protection against cell death and reduced ROS and mitochondrial membrane potential disruption	[167]
Cow’s milk	*Lactobacillus fermentum* LAB9 or *Lactobacillus casei* LABPC	BV2 cells	MTT assay, Griess reagent, and CD40 immunophenotyping	Decreased in NO level without affecting cell viability and no effect in CD40 expression	[168]
*Curcuma longa* L.	*Lactobacillus plantarum* K154 containing 2% (*w*/*v*) yeast extract	BV2 and C6 cells	MTT, NO, PGE2, and TNF-α assays	Prevented the cell death and inhibited PGE2 and NO production	[169]

* indicates isolated microorganism(s) from kimchi; TPC, total phenolic content; TFC, total flavonoid content; FRAP, ferric reducing ability of plasma; DPPH, 2,2-diphenyl-1-picrylhydrazyl; SOD, superoxide dismutase; MTT, 3-(4,5-dimethylthiazol-2-yl)-2,5-diphenyl tetrazolium bromide; AO/PI, acridine orange and propidium iodide; SEM, scanning electron microscopy; TEM, transmission electron microscopy; Tp73, tumor protein 73; BDNF, brain-derived neurotrophic factor; Aβ, amyloid-beta; Bax, Bcl-2-associated X protein; Bcl-2, B-cell lymphoma-2; ROS, reactive oxygen species; H_2_O_2_, hydrogen peroxide; LDH, lactate dehydrogenase; MAPK, mitogen-activated protein kinase; GPx, glutathione peroxidase; GSTs, glutathione S-transferases; CCK-8, cell counting kit 8; LPS, lipopolysaccharide; CREB, cAMP response element-binding protein; MMPs, mitochondrial membrane potentials; CD40, cluster of differentiation 40; NO, nitric oxide; PGE2, prostaglandin E2, TNF-α, tumor necrosis factor α; COX-2, cyclooxygenase-2; iNOS, nitric oxide synthase.
